# An improved 3D tetraculture system mimicking the cellular organisation at the alveolar barrier to study the potential toxic effects of particles on the lung

**DOI:** 10.1186/1743-8977-10-31

**Published:** 2013-07-26

**Authors:** Sebastian G Klein, Tommaso Serchi, Lucien Hoffmann, Brunhilde Blömeke, Arno C Gutleb

**Affiliations:** 1 Department Environment and Agro-biotechnologies (EVA), Centre de Recherche Public - Gabriel Lippmann, 41 rue du Brill, L-4422 Belvaux, Luxembourg; 2 Department of Environmental Toxicology, University of Trier, Universitätsring 15, Trier, Germany

**Keywords:** Nanoparticles, Inflammation, Oxidative stress, *In vitro* system, Coculture, Air-liquid-interface (ALI)

## Abstract

**Background:**

Exposure to fine and ultra-fine ambient particles is still a problem of concern in many industrialised parts of the world and the intensified use of nanotechnology may further increase exposure to small particles. Complex *in vitro* coculture systems may be valuable tools to study particle-induced processes and to extrapolate effects of particles on the lung. A system consisting of four different human cell lines which mimics the cell response of the alveolar surface *in vitro* was developed to study native aerosol exposure (Vitrocell™ chamber). The system is composed of an alveolar type-II cell line (A549), differentiated macrophage-like cells (THP-1), mast cells (HMC-1) and endothelial cells (EA.hy 926), seeded in a 3D-orientation on a microporous membrane.

**Results:**

The spatial distribution of the cells in the tetraculture was analysed by confocal laser scanning microscopy (CLSM), showing a confluent layer of endothelial and epithelial cells on both sides of the transwell. Macrophage-like cells and mast cells can be found on top of the epithelial cells. The cells formed colonies under submerged conditions, which disappeared at the ALI. To evaluate the response to oxidative stress, the dichlorodihydrofluorescein diacetate (DCFH-DA) assay was used together with 2,2’-azobis-2-methyl-propanimidamide-dihydrochloride (AAPH) as inducer of oxidative stress. The tetraculture showed less induction of reactive oxygen species (ROS) production after being treated with a positive control compared to the monocultures of EA.hy 926, THP-1 and HMC-1. Submerged cultures showed elevated ROS and IL-8 levels compared to ALI cultures. The Vitrocell™ aerosol exposure system was not significantly influencing the viability. Using this system, cells were exposed to an aerosol of 50 nm SiO_2_-Rhodamine NPs in PBS. The distribution of the NPs in the tetraculture after exposure was evaluated by CLSM. Fluorescence from internalized particles was detected in CD11b-positive THP-1 cells only.

**Conclusion:**

The system can be used in conjunction with a native aerosol exposure system and may finally lead to a more realistic judgement regarding the hazard of new compounds and/or new nano-scaled materials in the future. The results for the ROS production and IL-8 secretion suggest that submerged exposure may lead to an overestimation of observed effects.

## Background

Exposure to small ambient particles like particulate matter (PM; aerodynamic diameter <10 μm) is of high concern in many industrialised countries. Many studies indicate that continuous exposure to air pollution and to PM significantly increases morbidity and mortality related to respiratory and cardiovascular diseases [1]. The relationship between daily exposure to polluted air and augmented mortality became dramatically clear during the London fog episode in 1952 that was followed by a clear increase in mortality [2].

One possible explanation for the toxicity of atmospheric dust is that these particles can absorb pneumotoxic heavy metals as well as polycyclic aromatic hydrocarbons that can be found on their surfaces [3]. Particle bound transport is considered to be a fundamental pathway for the distribution of these toxic compounds in the environment [4]. Besides the larger particles with an aerodynamic diameter between 1 (PM_1_) and 10 (PM_10_) μm smaller particles of other size classes can also have detrimental effects on human health. During recent years the intensified use of nanotechnology led to the production of many new nanomaterials. The use of these materials may further increase exposure to ultrafine particles (aerodynamic diameter <100 nm) with potentially enforcing the risk for respiratory diseases. Nanomaterials and nanoparticles (NPs) (the first defined as a material with at least one dimension <100 nm and the latter as a material with all dimensions <100 nm) have become of primary interest for different kinds of industries. This downscaling enables the material to interact with the surrounding environment at a quantic level, opening the opportunity to generate material with new properties compared to the original bulk material. Despite a clear lack of knowledge on the toxicity of NPs, more than 1000 customer products already contain NPs (http://www.nanotechproject.org). NPs are supposed to have adverse effects on human health leading to an aggravation of pre-existing diseases, like asthma [5] and some reports suggest that the toxicity of PM_10_ is actually mainly linked to the ultrafine fraction [6]. Due to their small size, NPs can cross the alveolar barrier and affect the underlying cells or even enter the bloodstream thus causing damage in other parts of the body [7-10].

Among the mechanisms suggested to explain the adverse effects of particulates, the production of oxidative stress is likely of particular importance. Fine and ultrafine particles possess a high surface to mass ratio. In respect to this vast surface area per mass, the molecules that can be found on the surface can react more efficiently and in higher number with the surrounding environment, such as tissues, cellular membranes, etc. It is assumed that those surface molecules are in particular responsible for the observed oxidant capacity of particulates [11]. Oxidative stress plays an important role in respiratory diseases, such as allergic asthma and rhinitis [12]. The ability shown by some particles, such as diesel exhaust particles (DEP), carbon black or urban PM to produce reactive oxygen species (ROS) [13-16] strengthens the association between elevated exposure to particulates and exacerbations of lung disease and tissue damage [17], making the production of reactive oxygen species maybe the most important mechanism of particle-induced adverse effects.

Respiratory sensitization, as a consequence of the chronic exposure to particulates or chemicals, is a problem of high concern [18,19]. The evaluation of the toxic potential of new compounds, including chemicals and NPs present in many customer and personal care products, is an important task for which unfortunately, suitable *in vitro* and *in vivo* assays are still missing [20].

In this respect, complex *in vitro* coculture systems may be valuable tools to study pulmonary processes and further evaluate the effects of particles on the lung and human health [21]. The pulmonary system with almost 40 different cell types is highly heterogenous [22]. Even a state-of-the-art coculture system is still far from completely mimicking an *in vivo* tissue, but compared to monocultures, coculture systems allow cell-to-cell communications and regulations [23]. The possibility of spatial interaction between the cell types may change the observed effects and thereby result in a more realistic judgement regarding the hazardous potential of compounds, making coculture systems more relevant than monoculture models. Such models have a high potential to unravel some of the mechanisms involved, and will serve as a partial replacement for *in vivo* studies in the future.

An interesting *in vitro* approach combining A549 AT-II cells [24], THP-1 cells differentiated to macrophage-like cells [25,26], a human mast cell line (HMC-1 [27]) and EA.hy 926 endothelial cells [28] in coculture has been developed to assess the hazard of PM_10_ on the alveolar epithelium [29].

In this system endothelial cells were seeded in a transwell insert and a tripleculture consisting of A549 cells, differentiated THP-1 cells and HMC-1 cells was seeded inside the well of a multiwell plate. Both, the insert with endothelial cells and the tripleculture were cultivated in the same well to give the possibility to the cells to communicate with each other via soluble second messengers. This communication between endothelial cells and the cells that have the direct contact with particles may play an important role in the systemic effects of PM secondary after an inflammatory reaction. The aim of this study was to modify and to further improve this system so that it can be used in combination with the state-of-the-art aerosol exposure system (Vitrocell™ chamber). In contrast to the original system, where the actual tetraculture is divided into the part that is seeded in the multiwell plate (tripleculture: A549, THP-1, HMC-1), and the endothelial cells (EA.hy 926) seeded in the transwell insert, in our version the transwell plays the central role, as it serves as support for the complete tetraculture system. The coculture is designed in order to have a 3D-organisation of the cells, which closely resembles the *in vivo* histology of the alveolar barrier: endothelial cells are seeded on the basolateral side of a microporous membrane; epithelial cells together with the models for the innate immune system (mast cells and macrophage-like cells) are seeded in the apical compartment, cultivated at the ALI. The system offers possibilities to evaluate the inflammatory effects of NPs and PM via aerosol exposure at the ALI. The NPs or chemicals could be added to the upper compartment comprising the triculture. Eventually secreted second messengers can cross the microporous membrane and potentially affect the endothelial cells in the lower compartment.

In this paper we describe in detail our modified three-dimensional model of the alveolar barrier. The redesigned coculture system was optimised to be coupled to the Vitrocell™ system for the exposure of the cells under realistic conditions at the ALI, taking in consideration the presence of surfactant as an additional barrier *in vivo*. Due to interactions with medium components, such as serum proteins, the exposure of cells to particles under submerged conditions generally cannot properly reflect the exposure conditions of the alveolar epithelium *in vivo*[30]. A more realistic assessment of effects can be achieved by exposing cells at the air-liquid-interface to an aerosol of particles under controlled conditions [31-34], that would also allow better dosimetry [35].

## Results

### Structure of epithelial and endothelial cell layers on the microporous membrane

**Figure 1:**
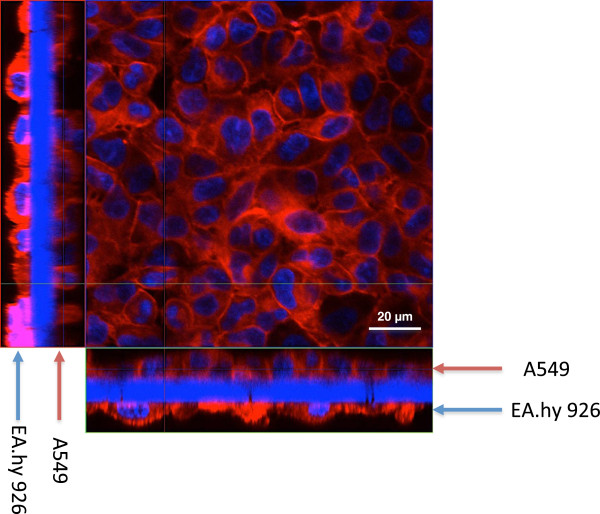
**Z-stack image series to evaluate the distribution of A549 and EA**.**hy 926 cells on opposite sides of a transwell insert.** The cells form a closed monolayer on both sides of the 1 μm transwell membrane. Cellular membranes are stained in red (cell mask deep red dye), nuclei in blue (DAPI). X–y projection with the respective side views (Magnification: 630x).

Transwell inserts (1 μm pore size) seeded with cells (A549 and EA.hy 926 in coculture) were stained with DAPI (blue) and cell mask deep red dye (red) and analysed by confocal laser scanning microscopy (CLSM). EA.hy 926 cells seeded on the basolateral side and A549 cells on the apical side of the transwell did not show any evidence of multilayer formation and the cells were distributed in a confluent monolayer. EA.hy 926 endothelial cells on the basolateral side of the membrane seemed to cover a larger surface area, compared to A549 cells and to have a flatter shape, while the A549 cells on the apical side appeared to be more cuboidal-shaped (Figure 1).

### Distribution of differentiated THP-1 cells and HMC-1 cells in the *in vitro* system

**Figure 2:**
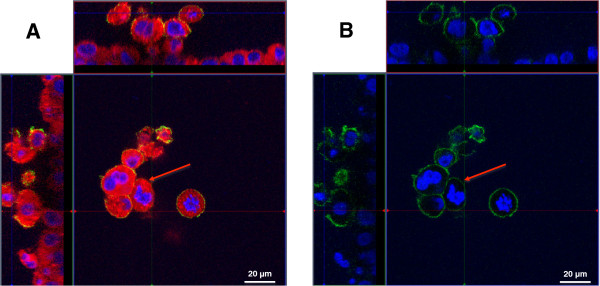
**Colonies of differentiated THP-1 cells on top of epithelial A549 cells. A**: Analysis of the distribution of THP-1 cells in the coculture system by a z-stack image series. THP-1 cells form colonies of several cells on top of A549 cells. THP-1 cells are stained with anti-CD11b-antibody (green); nuclei are counterstained with DAPI (blue). X–y projection with the respective side views (Magnification: 630x). **B**: Same image as shown in **A** but only with the channels for DAPI and CD11b. The red arrows indicate representative colonies (Magnification: 630x).

Differentiated macrophage-like THP-1 cells were seeded on top of A549 cells in transwell inserts. Cells were allowed to attach for 24 hours and then stained and the membranes were mounted. Cells were stained with DAPI (nuclei; blue), a fluorescent dye specific for the plasma membrane (red) and an antibody against CD11b (green) to highlight differentiated macrophages. The macrophage cells were triple positive, showing a signal for DAPI, plasma membrane and for CD11b. A549 cells did not express CD11b and a monolayer of cells can be observed (Figure 2A). In submerged conditions, once in contact with the epithelial cells, the differentiated THP-1 cells tend to form small colonies on top of the epithelial cell layer (Figure 2A and B). This effect was not observed in monocultures of THP-1 cells after differentiation.

**Figure 3:**
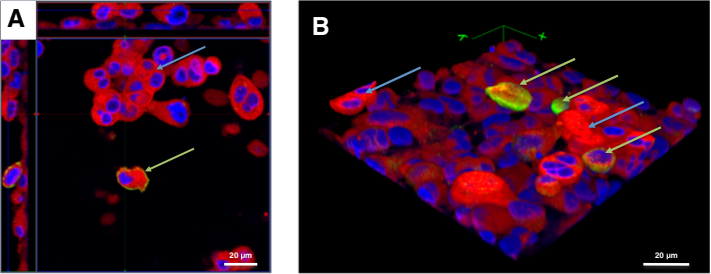
**Z-stack image series to analyse the distribution of THP-1 macrophages and HMC-1 in the tetraculture system present in the apical compartment of the insert.** The distribution of A549, differentiated THP-1, HMC-1 and EA.hy 926 cells in the tetraculture was analysed via CLSM. Cellular membranes are stained with cell mask deep red dye (red) and nuclei are stained with DAPI (blue); Macrophage-like cells are counterstained with an anti-CD11b-antibody. **A**: X–y projection with the respective side views. **B**: 3D reconstruction of the tetraculture based on the results of the z-stack from **A**. THP-1 (green arrows) and HMC-1 (blue arrows) cells are found on top of the epithelial cells. EA.hy 926 cells were not considered in the 3D reconstruction.

**Figure 4:**
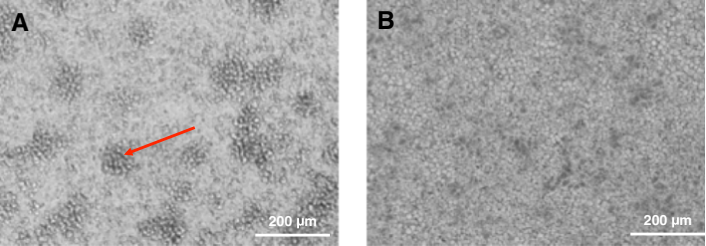
**Colonies of differentiated THP-1 cells and HMC-1 cells on top of epithelial A549 cells. A**: Brightfield image of THP-1 cells and HMC-1 cells that form colonies on top of A549 epithelial cells. The red arrow indicates a representative colony. **B**: Same transwell insert cultivated at the ALI without showing colonies. (Magnification: 200x).

A similar observation was made in the tetraculture system, composed of A549, differentiated THP-1, HMC-1, and EA.hy 926 cells. After seeding the HMC-1 cells into the tetraculture system, the originally floating HMC-1 cells disappeared from the culture medium and attached to the epithelial cell layer. The macrophage-like cells and mast cells can be found on top of the epithelial cells (Figure 3A and B). THP-1 cells are in direct contact with HMC-1 cells and they form heterogeneous colonies (Figure 3B). However, once the system was shifted to ALI conditions, the colonies that were observed under submerged conditions disappeared and the cells spread more equally on top of the epithelial cells (Figure 4A and B).

### Barrier qualities of the *in vitro* system

**Figure 5:**
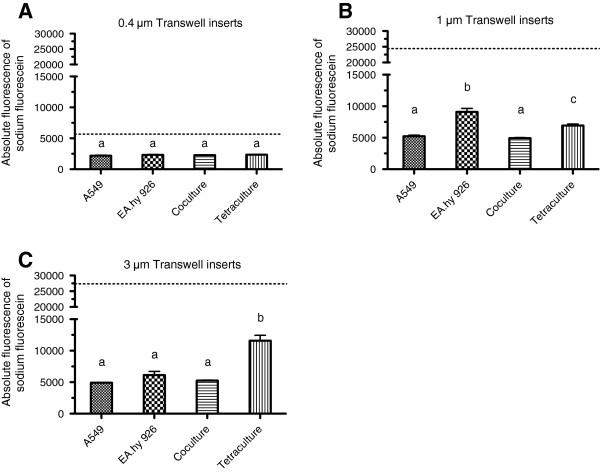
**Sodium fluorescein leakage for mono-, co-, and tetraculture in transwell inserts with 0.4, 1 and 3 μm pore size.** Medium containing 10 µg/mL of sodium fluorescein was administered to the apical compartment. Medium w/o sodium fluorescein was administered to the basolateral compartment and the leaked fluorescence was measured after 60 minutes incubation at room temperature in the dark. Results obtained for inserts with a pore size of 0.4 μm, 1 μm and 3 μm are given in **A**, **B** and **C**, respectively. The dotted line represents the leakage of transwell inserts w/o cells. Data represents the mean of four independent transwell inserts ± SEM. Groups that are sharing the same letters are not significantly different (*P* > 0.05).

A549 in monoculture, EA.hy 926 in monoculture, A549 plus EA.hy 926 in coculture and the tretraculture were exposed for 60 minutes to a fluorescent tracer solution in the apical compartment and the fluorescence leakage was measured in the basolateral compartment. Inserts of different pore sizes (0.4, 1 and 3 μm) were compared as well (Figure 5A, B and C). The 0.4 μm inserts showed a great ability to block the fluorescent tracer solution, regardless of the presence or absence of cells (Figure 5A). In inserts with 1 and 3 μm pore size, the presence and the composition of the cell layers influences the amount of leaked fluorescence. The overall leakage in an empty 0.4 μm transwell insert (w/o cells) was four times less compared to inserts with 1 and 3 μm pores (compare empty inserts in Figure 5A, B, and C). For cell cultures prepared in 0.4 μm inserts, no statistically significant differences were observed (Figure 5A). When 1 μm inserts are used, the monoculture of EA.hy 926 shows with 9103 ± 545 significantly the highest leakage (*P* < 0.05). When A549 and EA.hy 926 cells are in coculture, the leakage (4944 ± 93) is slightly lower than in the A549 monoculture (5241 ± 161). In the tetraculture system with A549, EA.hy 926, differentiated THP-1 and HMC-1 cells, the leakage is with 6339 ± 249 again higher than for the A549 monoculture and the coculture of A549 and EA.hy 926 but still significantly lower than for the monoculture of EA.hy 926 (*P* < 0.05) (Figure 5B). In transwell inserts with 3 μm pores, the amount of leaked fluorescence for A549 monoculture is 4928 ± 57 and for EA.hy 926 in monoculture 6134 ± 572. The coculture of A549 and EA.hy 926 cells shows a fluorescence leakage of 5250 ± 84. The differences between A549 and EA.hy 926 cells in monoculture and the corresponding coculture were not significant. In these inserts the tetraculture shows the highest amount of leaked fluorescence with 11584 ± 865 (Figure 5C).

The same phenomenon was observed when the accessibility of resazurin for cells grown in transwell inserts of different pore sizes was evaluated. For cells cultivated in transwell inserts with a pore size of 0.4 μm, resazurin on the opposite side of the membrane was not accessible for conversion. However, when transwell inserts of 1 and 3 μm pore size were tested, the resazurin became accessible to the cells (Additional file 1).

### Behaviour of the tetraculture in response to an oxidative stress inducer

The production of ROS as a response to the exposure to small particulates is an important effect of particles and ROS production by particles has also been linked to their adverse effects on the cardiovascular system. To see how differently our cultures respond to an oxidative stimulus, monocultures, cocultures, and tetracultures were exposed to 20 mM 2,2’-azobis-2-methyl-propanimidamide-dihydrochloride (AAPH) for 2 h after which the oxidation of the dye DCFH-DA as indicator of ROS production was measured.

**Figure 6:**
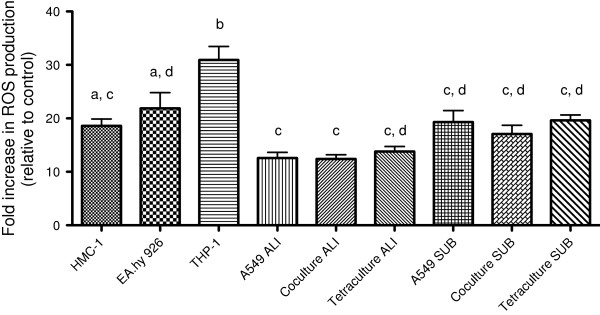
**DCFH-DA assay to analyse the behaviour of the different cultures in response to oxidative stress.** Cultures were preloaded with DCFH-DA dye for 45 min and subsequently exposed to 20 mM AAPH in medium for 2 h. The oxidation of DCFH-DA was measured as an augmentation of green fluorescence. The increase in ROS production was compared to cells that were treated with medium without AAPH. Data represents the mean of at least four independent transwell inserts ± SEM. Groups that are sharing the same letters are not significantly different (*P* > 0.05).

The differentiated THP-1 cells showed the highest fold increase in ROS production (30.93 ± 2.521; *P* < 0.05) for all the cultures tested. Among the monocultures, the response of HMC-1 and EA.hy 926 cells was comparable with a fold increase of 18.59 ± 1.29, respectively 21.89 ± 2.9. A549 cells were cultivated at the ALI and under submerged conditions and then exposed to AAPH. The cells cultivated at the ALI showed the lowest fold increase of all tested monocultures (12.6 ± 1.03; *P* < 0.05 when compared to EA.hy 926 and THP-1). The fold increase of ROS production for the coculture of A549 and EA.hy 926 cells at the ALI as well as the fold increase for the tetraculture at the ALI was comparable to the results obtained with A549 cells alone. Cultures that were grown under submerged conditions showed the tendency to exhibit higher ROS levels compared to cultures grown at the ALI. Also here, the monoculture of A549 cells, the coculture and the tetraculture showed comparable results (Figure 6).

### Secretion of pro-inflammatory cytokines

*In vivo*, cells have the possibility to communicate and to influence each other. This crosstalk between cells also influences the production of mediators. Here we measured the secretion of the pro-inflammatory cytokines IL-1β, IL-6, GM-CSF, IL-8 and TNF-α to evaluate possible differences in the cytokine production pattern between our cultures in the acute phase after being treated with AAPH for 2 h. The levels of the cytokines were compared between A549 in monoculture, the coculture of A549 and EA.hy 926 cells and the tetraculture. In addition we also compared submerged and ALI culture.

**Figure 7:**
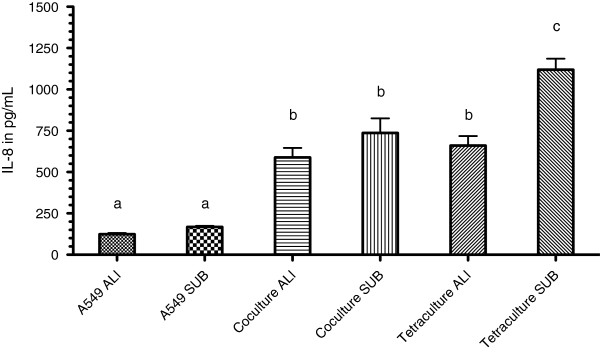
**Evaluation of the levels of IL-8 after treatment with AAPH.** Cultures were exposed to 20 mM AAPH in medium for 2 h. Afterwards samples of the undernatant were collected and analysed to evaluate the amounts of IL-8. Data represents the mean of at least four independent transwell inserts ± SEM. Groups that are sharing the same letters are not significantly different (*P* > 0.05).

When exposed to 20 mM AAPH for 2 h, A549 cells in monoculture secreted 124.7 ± 7.4 pg/mL submerged and 168 ± 5.8 pg/mL IL-8 when cultivated at the ALI (n ≥ 4). The results obtained for A549 cells alone were the lowest compared to the coculture and the tetraculture (*P* < 0.05). The coculture showed significantly elevated IL-8 levels of 588 ± 58 pg/mL (ALI), respectively 736 ± 88 pg/mL (submerged) compared to A549 cells (P < 0.05). The response of the tetraculture at the ALI with 660 ± 57.49 pg/mL IL-8 was comparable to the response of the coculture. The tetraculture cultivated under submerged conditions showed the highest IL-8 levels with 1118 ± 66.86 pg/mL compared to all other cultures (*P* < 0.05) (Figure 7).

The comparison between submerged culture and ALI culture shows that the IL-8 seretion is slightly elevated for A549 cells and the coculture (not significantly) and dramatically increased for the tetraculture with almost a two-fold increase when compared to the submerged counterpart (*P* < 0.05) (Figure 7).

Levels for IL-1β, IL-6, TNF-α and GM-CSF were below the limit of detection (data not shown).

### Cell viability after aerosol treatment with the Vitrocell™ exposure system

**Figure 8:**
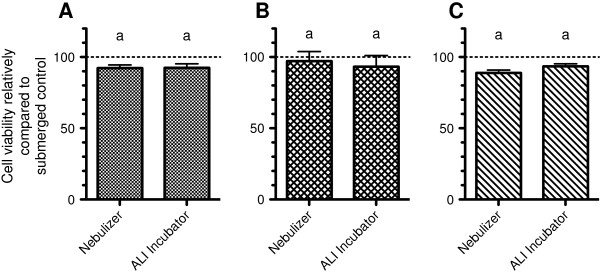
**Viability of cultures exposed to an aerosol of PBS by using the Vitrocell™ aerosol exposure system.** Cultures were exposed to an aerosol of PBS for 30 min using the Vitrocell™ aerosol exposure system. **A**: A549 monoculture; **B**: Coculture of A549 and EA.hy 926 cells; **C**: Tetraculture. The dotted line represents the viability of cells kept under submerged conditions to which the ALI samples were compared. Data represents the mean of four independent transwell inserts ± SEM. Groups that are sharing the same letters are not significantly different (*P* > 0.05).

The effect of exposure conditions present in the Vitrocell™ aerosol exposure system on cellular viability was evaluated by exposing different cell cultures to an aerosol of PBS and ambient sterile filtered air for 30 min. The cultures (A549 in monoculture, in coculture with EA.hy 926 cells and in tetraculture with EA.hy 926, THP-1 and HMC-1) were kept under ALI conditions in the incubator for 24 h and then exposed. Immediately after exposure, the integrity of the cell layers was verified by light microscopy and no visible alterations were observed (data not shown). Subsequently the cultures were placed back into the incubator and the cellular viability was measured 24 h later. Cell viability was compared to cultures under submerged conditions and cultures kept in the incubator at the ALI for 48 h (Figure 8). Compared to cells under submerged conditions, the viability was not significantly reduced (less than 12%) for ALI cultures. No significant differences between cells exposed in the aerosol chamber and cells incubated at ALI conditions in the incubator were observed.

### Behaviour of macrophage-like cells in submerged exposure and at the air-liquid-interface

*In vivo*, alveolar macrophages are efficiently intercepting particles and foreign materials in the alveolar region, contributing to clearance and defence of the alveolar region. The ability of the macrophage-like cells to intercept particles in the tetraculture was evaluated.

**Figure 9:**
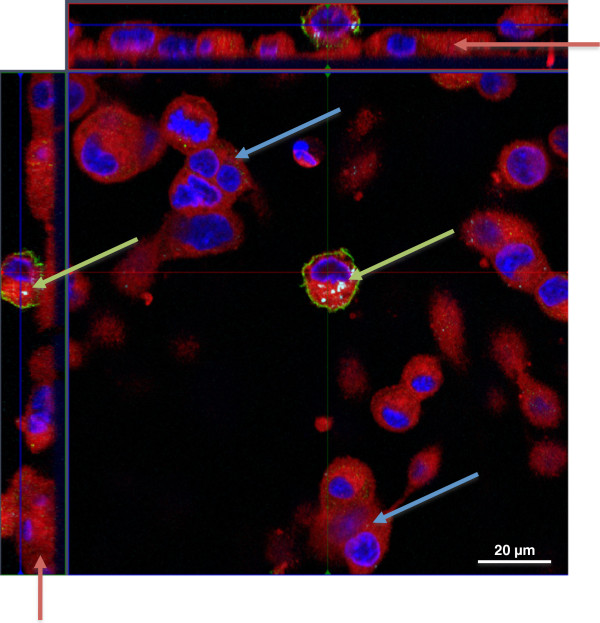
**Z-stack image series to analyse the phagocytic activity of THP-1 macrophage-like cells in the tetraculture present in the apical compartment of the system.** Tetracultures of A549, differentiated THP-1, HMC-1 and EA.hy 926 were exposed to cell culture medium containing 10 mg/L of 50 nm SiO_2_-Rhodamine particles for 24 h. SiO_2_-Rhodamine particles distribution was analysed via CLSM. Cellular membranes are stained with cell mask deep red dye (red) and nuclei are stained with DAPI (blue). Macrophage cells are counterstained with an anti-CD11b-antibody (green). Fluorescence from ingested SiO_2_-Rhodamine particles was detected in differentiated THP-1 cells situated on top of the A549 cells (green arrows) but not in A549 (red arrows) or HMC-1 (blue arrows). The image shows an x–y projection with the respective side views.

The tetraculture was incubated for 24 h with 50 nm SiO_2_-Rhodamine particles suspended in tetraculture cell medium (10 mg/L) with 1% serum. Afterwards, cells were fixed and stained with DAPI, cell mask deep red dye and anti-CD11b-antibody, and analysed via CLSM. Signals for internalised SiO_2_-Rhodamine NPs were detected inside of CD11b-positive macrophage-like THP-1 cells. In the A549 cell layer as well as in the HMC-1 cells, no internalised SiO_2_-Rhodamine NPs could be detected (Figure 9).

In analogous conditions to the submerged exposure, tetracultures were exposed to an aerosol of PBS containing 50 nm SiO_2_-Rhodamine particles. The aerosol was generated by a pneumatic nebulizer (AGF 2.0 PALAS, Karlsruhe, Germany) and was delivered to the modules through a trumpet device at a flow rate of 5 ± 0.1 mL/min/module for a defined time of exposure (30 minutes). The concentration of the particle suspension in the reservoir of the AGF 2.0 nebulizer was 1 g/L.

**Figure 10:**
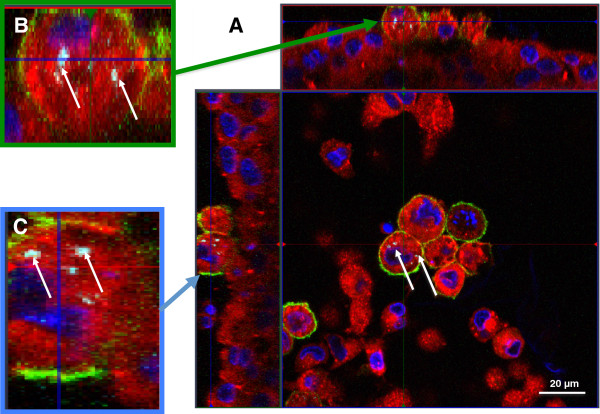
**Z-stack image series to analyse the phagocytic activity of THP**-**1 macrophages in the tetraculture present in the apical compartment of the system after exposure to 50 nm SiO**_**2**_**-Rhodamine nanoparticles with the Vitrocell™ system.** Tetracultures of A549, differentiated THP-1, HMC-1 and EA.hy 926 cells exposed to an aerosol of 50 nm SiO_2_-Rhodamine particles for 30 minutes using the Vitrocell™ aerosol exposure system. Afterwards cells were fixed, labelled and the distribution of SiO_2_-Rhodamine particles was analysed via CLSM. Cellular membranes stained with cell mask deep red dye (red) and nuclei with DAPI (blue) are shown. Macrophage-like cells are counterstained with an anti-CD11b-antibody. Signals of ingested SiO_2_-Rhodamine particles were detected in differentiated THP-1 cells (white arrows), sitting on top of the A549 cells but not in A549 or HMC-1 cells. **A**: The image shows an x–y projection with the respective side views. **B** and **C** show a macrophage-like cell from **A** in a higher magnification.

After exposure, the cells were allowed to recover for 48 h before fixation and staining. Inserts were stained with DAPI, cell mask deep red dye and CD11b and analysed via CLSM. Signals for internalised SiO_2_-Rhodamine NPs were detected inside of CD11b-positive macrophage-like THP-1 cells. In the A549 cell layer as well as in the HMC-1 cells and the endothelial layer (not shown), no internalised SiO_2_-Rhodamine NPs could be detected (Figure 10A, B and C).

## Discussion

The total number of *in vitro* approaches reported to be available for respiratory toxicology is relatively high. However, the majority of these *in vitro* systems are based on monocultures of cells derived from different regions of the respiratory system [21]. For some scientific questions, such as the induction of endothelial inflammation by particles, the use of a monoculture is not appropriate, since the direct exposure of endothelial cells to particles is in most cases poorly justified [36]. A possibility to address this issue is to use a multi-layered system which combines epithelial cells, immune cells and endothelial cells together [37,38]. Another limitation present in many systems currently used is the submerged exposure of cells. Such a system does not properly predict the hazard of the tested material, especially particles, since it has been shown that the exposure route itself may have a crucial impact on the results [39].

In the original version of the system established by Alfaro-Moreno and coworkers [29], the triculture of A549, THP-1 and HMC-1 cells was located at the bottom of a cultivation well and endothelial cells were seeded in a transwell insert in the same well [29]. Triculture and endothelial cells were physically separated by the microporous membrane and by a layer of medium, which does not resemble the histology of the tissue *in vivo* (Additional file 2). In our modified version the triculture of A549, THP-1 and HMC-1 was shifted from the bottom of the multiwell plate into the transwell insert and EA.hy 926 endothelial cells were moved from the inside of the transwell insert to the basolateral side of the membrane in order to better reflect the *in vivo* anatomy of the alveolar region. The system contains differentiated THP-1 cells and HMC-1 cells as models for the innate immune system together with A549 epithelial cells and this allows the study of inflammatory mechanisms in the alveoli *in vitro*.

Modifying the system initially described [29] a modification was presented which included a 3D-orientation, having a layer of primary endothelial cells (HUVECs) on the basolateral side of the transwell and epithelial cells (A549) in the apical compartment [37,38]. This system has been used to demonstrate that the indirect exposure of endothelial cells leads to the activation of the endothelial cells, to which immune cells (U937), but also cancer cells (MCF-7) can adhere [37,38]. This approach demonstrates the importance of cellular crosstalk between epithelial and endothelial cells. Similar experiments are conceivable, by using the tetraculture system presented in this publication to study the response of the endothelial cells after native aerosol exposure in presence or absence of the models for the innate immune system.

Furthermore the ratio of A549, THP-1 and HMC-1 cells was optimized in order to reproduce a physiologically relevant cell density and distribution [29]. In the alveolar model presented in this publication, the seeding ratios are most likely not resembling the *in vivo* condition, as there might be an overrepresentation of monocytes and mast cells, compared to endothelial and epithelial cells. The rationale for this seeding ratio is mainly based on methodological arguments. Protocols to separate the different cell types of the tetraculture after exposure in order to study the individual responses, require a higher number of cells. The same is true when applying -omic techniques, such as transcriptomics and proteomics, that require higher quantities of cells. In order to reduce the technical complexity of such experiments, we decided to increase the number of immune cells present in the system.

The transwell insert itself is one of the key factors of an ALI exposure system. It allows growing the cells in contact to the ambient air above and receiving nutrients from the medium below.

These inserts are available with different pore sizes and densities, and these parameters may influence cellular growth and availability of nutrients as well as soluble second messengers. Not all NPs can efficiently pass through a cell-free 0.4 μm transwell insert [40]. In general, particles with a size below 100 nm should be able to pass a 400 nm pore easily; however, this depends not only on the size but rather on the surface chemistry of the NP, resulting in electrostatic changes and different hydrodynamic diameters [40,41]. Therefore inserts of different pore sizes from the same supplier were tested in order to choose the most appropriate insert as a support for the 3D tetraculture system. In our setup, the viability of cultures in 0.4 μm inserts was generally lower compared to cultures grown in 1 and 3 μm inserts (see Additional file 3). In addition, in A549 cultures grown at the ALI in 3 μm pore size membranes a considerable amount of liquid leached from the lower compartment into the upper compartment, while in 1 μm pore size inserts the quantity of liquid appearing in the upper compartment was negligible.

The tightness of the epithelial barrier in the system was analysed by adding a sodium fluorescein tracer-solution to the apical compartment after which the appearing fluorescence in the basolateral compartment was measured. An epithelium is considered to be efficiently sealed if the amount of leaked fluorescence within one hour is less than 1% of the initial concentration [40,42]. In our setup, transwell inserts with 0.4 μm pores were significantly blocking the transport of tracer molecules across the membrane. This effect is larger than the ability of the different cultures to block and influence the liquid exchange. The tracer molecule used is small and is used as a reference for the passage of nutrients and second messengers through membranes. The tetraculture system showed for the 1 and 3 μm inserts significantly higher barrier permeability than other culture variations. Only the EA.hy 926 cells in monoculture in 1 μm inserts showed a higher amount of leaked fluorescence. The accessibility of resazurin for the cells through the membrane in dependence on the pore size is consistent with the results observed for sodium-fluorescein (see Additional file 1).

The high permeability of endothelial cells can be explained by their *in vivo* function. Endothelial cells are found on the inner side of blood vessels (intima). Depending on the type of blood vessels, these cells have more or less tight and gap junctions resulting in different permeability [43].

The presence of the model cells for the innate immune system was shown to have a significant impact on the behaviour of A549 epithelial cells, which resulted in decreased transepithelial electrical resistance (TEER) [44]. The expression and presence of functional tight junctions is mainly responsible for high TEER values and a reduction can be considered to be followed by higher paracellular permeability [45]. The presence of functional tight junctions expressed by A549 cells, sealing the epithelium and allowing a high TEER is controversially discussed [21] and under assay conditions, no significant differences in TEER were observed (Additional file 4). Nevertheless, THP-1 and HMC-1 cells in the tetraculture seem to have an impact on the tightness of the cell layer, which resulted in a higher permeability of the tracer solution used. This is in line with the observations made by Lehmann and coworkers [44]. Although A549 cells provide certain benefits when used in an *in vitro* model, such as the production and secretion of surfactant (Additional files 5, 6 and 7) [46,47], the ability to secrete pro-inflammatory mediators and their phase I and phase II metabolic activities [48], the lack of tight junction is a disadvantage for some experimental endpoints. Especially for studies regarding the uptake efficiency of drug candidates or other translocation studies the use of a system based on primary epithelial cells may be more appropriate [49]. However, the use of primary cells brings additional technical problems, such as rapid dedifferentiation potential, donor-to-donor variation, limited passage number, etc.

Cell distribution on the two sides of the transwell membrane for A549-EA.hy 926-cocultures was analysed by CLSM. The results show a confluent monolayer of epithelial A549 cells in the apical compartment and a confluent monolayer of endothelial EA.hy 926 cells on the basolateral side of the membrane. A549 cells show a more polarized cuboidal shape [50], whereas EA.hy 926 cells are flatter and cover a larger surface area than A549 cells. Contrary to published reports [51], A549 cells did not form multi-layers in our experimental conditions even when cultured for several days.

The alveolar region lacks a mucociliary escalator, and the clearance of foreign material is mainly restricted to the macrophage phagocytosis [52]. *In vivo*, this population of alveolar macrophages (AM) is efficiently ingesting bacteria and also intercepts particles. AMs are the key players in alveolar clearance and show generally a higher phagocytic activity than macrophages located in other tissues [53,54]. The fate of particles in the alveolar region depends on their physico-chemical properties. Some kinds of NPs can be translocated through the epithelium and reach the bloodstream or other organs [55]. THP-1 cells are used as a surrogate for macrophage-like cells. Upon differentiation stimuli, they differentiate into mature macrophage-like cells and are known to show a considerable phagocytic activity [56].

The macrophage-like THP-1 cells showed the tendency to form colonies once seeded on top of the epithelial layer in coculture. During the differentiation of THP-1 in monoculture, cells did not form such colonies, but rather stayed attached as a single cell layer to the supporting material. A similar observation was made for HMC-1 cells. HMC-1 cells in monoculture, without stimulation, floated and only a few cells attached to the support. In the tetraculture, almost all floating HMC-1 cells disappeared and attached to the epithelial layer. Furthermore it was observed that THP-1 and HMC-1 cells formed heterogeneous colonies on top of the A549 epithelial cells, which is not properly reflecting the *in vivo* situation. However, when the cultures were switched to the ALI conditions the colonies disappeared and the differentiated macrophage-like cells and the HMC-1 cells spread more equally on top of the A549 epithelial cells.

The functionality of HMC-1 to produce histamine and the differentiation state of macrophage-like THP-1 cells were evaluated (Additional files 8, 9A and B). In addition the activity of THP-1 cells to act as particle-intercepting cells was studied by exposing tetracultures to 50 nm SiO_2_-Rhodamine NPs under submerged conditions and at the ALI. Signals for SiO_2_-Rhodamine particles were detected inside of THP-1 cells but not in other cell types of the tetraculture. Principally, a single 50 nm SiO_2_-Rhodamine particle would be far beyond the detection range of any confocal microscope. However, by the use of CLSM together with digital image restoration technologies, even weak intracellular signals of NPs could be detected [57]. The signals detected in THP-1 cells were likely the result of a high number of internalized particles that are stored as agglomerates in sub-cellular compartments, possibly endosomes or lysosomes [58].

This is in line with published results showing that the strongest signals of particles were detected in phagocytotically active cells, such as macrophages and dendritic cells [57]. The use of aerosol exposure systems is more relevant than the exposure under submerged conditions: interactions of particles with molecules present in cell culture media can alter their properties [30], potentially leading to misevaluating the hazard of these particles. In addition, it was recently shown that the route of exposure influences the toxicity of particules, leading to a potential underestimation of effects in submerged exposure [59].

Several NPs are known to induce oxidative stress [60-64]. The production of the involved ROS can either be direct or indirect, depending on the composition and the surface chemistry of the nanomaterial [65]. Oxidative stress is considered to be one of the major mechanisms of how particles affect cells, tissues and organs [65,66]. The behaviour of A549 cells in monoculture, the coculture between A549 and EA.hy 926 cells and the tetraculture at the ALI and under submerged conditions in response to an oxidative stimulus was evaluated. Compared to the monocultures of EA.hy 926, THP-1 and HMC-1, the fold increase of ROS in the tetraculture is lower. THP-1 cells were found to have the highest fold increase in ROS production (*P* < 0.05). Although the differences between cells grown at the ALI and cells kept under submerged conditions were not significantly different, ALI cultures showed the tendency to exhibit lower ROS levels. This may be due to adaption to the higher amount of oxygen and radicals at the ALI, making the cells less sensitive to the effects of ROS. This is in line with the IL-8 levels. Although there was no on-going inflammation, A549 cells and the monoculture showed the tendency to produce more IL-8. The tetraculture at the ALI secreted only ≈ 50% of the IL-8 amounts estimated in the submerged tetraculture. In addition it is interesting to note that there was no synergistic effect in the tetraculture although the monocultures were efficiently responding to the oxidative stress inducer. One possible explanation for the similar levels of ROS in the A549 monoculture, the coculture and the tetraculture is the high production of glutathione by A549 cells, which is possibly depleting efficiently the radicals and may exhibit a protective effect [67].

We can conclude that the pro-oxidative conditions to which the monoculture, coculture and tetraculture were exposed to are generally well tolerated by the cells and, overall, not high enough to induce a consistent inflammatory response. However, it seems that the cultures grown at the ALI are able to better tolerate the treatment and do react in a more efficient way to ROS stimuli, as demonstrated by the reduced IL-8 amounts.

## Conclusions

An improved tetraculture system, representing the alveolar barrier, has been carefully characterised and adapted for native aerosol exposure. The cultivation of cells at the air-liquid-interface is a prerequisite for the administration of PM and NPs via aerosol. Resembling the *in vivo* histology of the alveolar barrier, the endothelial cells are grown on the basolateral side of the microporous membrane; epithelial cells and the models for the innate immune system are grown within the transwell insert. This setup ensures a 3D-orientation similar to the organisation of the alveoli *in vivo*. Inserts with pores of 1 μm were found to be the most appropriate support for our model system, since they ensure the optimal exchange of nutrients between compartments. Submerged cultures showed elevated levels in ROS production compared to ALI cultures.

The macrophage-like THP-1 cells act as particle-intercepting cells and form heterogeneous colonies with mast cells (HMC-1) in the *in vitro* system as shown by CLSM.

Our results demonstrate that the proposed system is behaving in a physiological way and has characteristics that resemble the *in vivo* organisation of the alveolar region. The macrophagic component of our model is actively and efficiently intercepting particles both in submerged conditions and exposed to an aerosol at the air-liquid-interface.

Overall the use of the tetraculture system may lead to a more realistic judgement regarding the hazard of new compounds in the future.

## Materials and methods

### Reagents

All reagents, unless otherwise specified, were purchased from Sigma Chemical (Deisenhofen, Germany). Cell culture media were purchased from Invitrogen (the Netherlands), fetal bovine serum (FBS Gold) was obtained from PAA (Paschl, Austria). SiO_2_-Rhodamine (50 nm) particles were purchased from Corpuscular (Cold Spring, NY, USA).

### Cell culture

The human cell lines A549 [24], THP-1 [25] and EA.hy 926 [28] were obtained from the American Type Culture Collection (Manassas, VA, USA). HMC-1 [27] cells were kindly provided by J.H. Butterfield, Mayo Clinic (Rochester, MN, USA). The cells were cultured using different media (Table 1).

**Table 1:** **Medium conditions for mono**- **and cocultures**

**Monocultures**			
**Cell Line**	**Medium**	**Serum**	**Supplements**
**A549**	Dulbecco’s Modified Eagle’s Medium (DMEM)	10% (v/v) Fetal Bovine Serum Gold	
**THP-1**	Roswell Park Memorial Institute (RPMI) 1640		25 mM HEPES; 50 μM β-mercaptoethanol
**HMC-1**	Iscove’s Modified Dulbecco’s Medium (IMDM)		25 mM HEPES; 1,2 mM α-thioglycerol
**EA.hy 926**	Dulbecco’s Modified Eagle’s Medium (DMEM)		25 mM HEPES
**Tetraculture**			
75% HEPES-buffered DMEM; 15% RPMI; 10% IMDM		10% (respectively 1%) (v/v) Fetal Bovine Serum Gold	25 mM HEPES

Unless further mentioned, mono- and cocultures were handled in the absence of antibiotic agents. All media contained Glutamax instead of L-glutamine.

Cells were seeded at specified densities (cells/cm^2^) (Table 2) on BD Falcon cell culture inserts (surface area of 4.2 cm^2^; 0.4, 1 and 3 μm pore size; high pore density PET membranes for 6-well plates; BD Biosciences, Basel, Switzerland) and grown until confluency. Inserts were placed in BD Falcon tissue culture plates (6-well plates; BD Biosciences) with 2 mL medium in the upper and 2 mL in the lower compartment. Cells were grown in T75 flasks and trypsinized twice a week. Medium (inserts and cell culture flasks) was changed every other day. Cells were maintained in a humidified atmosphere with 5% CO_2_ at 37°C and tested regularly for contamination by mycoplasma.

**Table 2:** Seeding densities for the different mammalian cell lines

**Cell line**	**Seeding density per cm**^**2**^
**A549**	1.2 x 10^5^
**EA.hy 926**	2.4 x 10^5^
**THP-1**	2.4 x 10^5^
**HMC-1**	1.2 x 10^5^

### Differentiation of THP-1 cells into macrophage-like cells

Human THP-1 cells (human acute monocytic leukemia cell line) [25] were grown in RPMI 1640 media containing 10% (v/v) FBS Gold (PAA, Paschl, Austria). Differentiation was achieved by resuspension of THP-1 cells at 4 × 10^5^ cells/mL in growth medium with addition of phorbol-12-myristate-13-acetate (PMA; 20 ng/mL) and incubation over night at 37°C and 5% CO_2_[56]. PMA was prepared as a stock solution (10 mg/mL) in ultrapure absolute ethanol. Stocks were kept at −20°C in the dark. Differentiated THP-1 cells were rinsed with PBS and detached by using accutase in order to harvest them. To remove traces of PMA, detached cells were centrifuged and washed twice with PBS. The upregulation of CD11b as a marker for mature macrophage cells [26] was evaluated by confocal laser scanning microscopy (CLSM) (see cell labelling and fixation).

### Tetraculture

**Figure 11:**
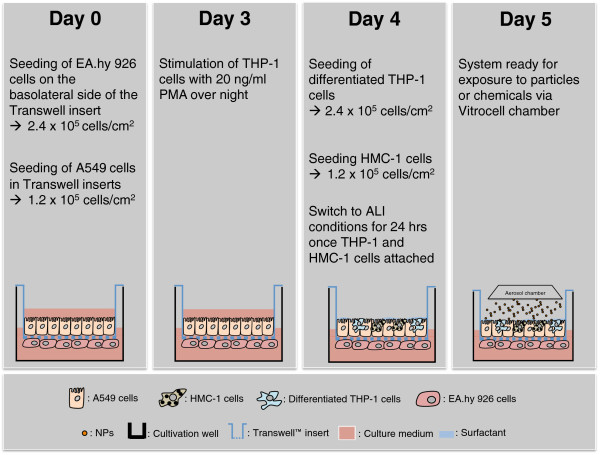
Workflow to setup an aerosol exposure experiment with the tetraculture system.

EA.hy 926 endothelial cells were seeded on inverted transwell inserts (2.4 × 10^5^ cells/cm^2^; BD Falcon inserts, 1 μm pore size; Additional file 10). Upon attachment on the basolateral side of the transwell insert the plate with the transwell inserts was turned back to its original orientation (Additional file 10, Step 4) before the A549 cells were seeded inside the transwell. Epithelial and endothelial cells were grown for three days at 37°C and 5% in a humidified incubator. On day 3 THP-1 cells were stimulated to differentiate into macrophage-like cells by addition of PMA. On day 4, THP-1 cells and HMC-1 cells were added into the inserts with A549 and EA.hy 926 cells to complete the tetraculture system. The medium for the complete tetraculture contained only 1% FBS to avoid extensive proliferation of HMC-1 cells. THP-1 cells and mast cells were found to be attached on the epithelial layer after 4 hrs. The attachment of the cells was verified by light microscopy. Upon attachment the medium was removed from the upper compartment and the tetraculture was cultivated at the ALI for 24 h prior to exposure (Figure 11).

### Transepithelial transport of sodium fluorescein

A solution of sodium fluorescein (10 µg/mL in medium) was added into the apical compartment and medium without sodium fluorescein was added into the lower compartment. The cultures were incubated for 60 min at room temperature in the dark. Empty inserts without cells served as a control to evaluate the maximum leakage. After 60 min, the inserts were carefully discarded and samples were taken from the basolateral compartment. The fluorescence of sodium fluorescein was measured with a multi-mode microplate reader (ex: 495 nm; em: 519 nm; Biotek, Germany).

### Adaptation of the DCFH-DA assay for oxidative stress for the use with transwell inserts

2,2’-azobis-2-methyl-propanimidamide-dihydrochloride (AAPH; Merck), a water-soluble azo-compound, which is commonly used in studies of lipid peroxidation, was chosen as positive control for oxidative stress. Working solutions were prepared freshly in cell culture medium before use. The evaluation of ROS production was performed by the use of dichlorodihydrofluorescein diacetate (DCFH-DA) dye. DCFH-DA enters the cell and the diacetate is cleaved, which results in the trapping of the dye inside the cell. In the presence of reactive oxygen species (ROS) the dye is oxidized and emits green fluorescence, which is directly proportional to the amount of ROS [68].

DCFH-DA was suspended in cell culture medium (100 μM) and cells were preloaded with the dye for 45 min at 37°C and 5% in the dark. After the preloading step the cells were rinsed with PBS to remove the non-internalized DCFH-DA and immediately exposed to the compound of interest. Fluorescence reading was done with a multi-mode microplate reader (ex: 485 nm, em: 528 nm; Biotek, Germany). Empty inserts did not emit significant fluorescence at the above-mentioned wavelengths.

### Cytokine measurements

Single cultures, cocultures and tetracultures were exposed to 20 mM of AAPH for two hours. After treatment, aliquots from the undernatant were taken and the amounts of GM-CSF, IL-1 β, IL-6, IL-8 and TNF-α were analysed. Quantification was performed on a Luminex 100™ (Luminex Oosterhout, the Netherlands) using the inflammatory cytokine human magnetic 5-Plex Panel produced by Invitrogen (Invitrogen, Leusden, the Netherlands) following manufacturer’s instruction.

### Cell labelling and fixation

The cells were washed twice in PBS and fixed for 15 min at room temperature in 4% formaldehyde in PBS (v/v). Fixed cells were incubated for 30 min with 10% bovine serum albumin (BSA) in PBS (w/v) to block unspecific bindings. After blocking, cells were incubated with primary and secondary antibodies for 60 min each at room temperature in the dark.

Antibodies were diluted in immunostain enhancer (Thermo Scientific, Belgium) as follows: rabbit anti-human CD11b 1:200 (clone EPR1344; Novus Biologicals, UK), goat anti-rabbit dylight 488 1:2000 (AS09 633; Agrisera, Vännas, Sweden). Nuclei were counterstained with 4’ ,6’-diamidino-2-phenylindole (DAPI) and cellular membranes were stained with cell mask deep red (C10046; Invitrogen, Leusden, the Netherlands).

### Confocal microscopy and image restoration

A Zeiss LSM 510 Meta with an inverted Zeiss microscope (Axiovert 200 M, Lasers: HeNe 633 nm, HeNe 543 nm, Ar 488 nm and Diode 405 nm; Zeiss, Jena, Germany) was used. Image processing and visualization was done using the Zeiss Software ZEN 2011 and ImageJ (http://rsbweb.nih.gov/ij/).

### Aerosol exposure

The Vitrocell aerosol exposure device (Vitrocell, Waldkirch, Germany) (Additional file 11) was used for dynamic delivery and exposure of cells to aerosolized PBS and to PBS containing 50 nm SiO_2_-Rhodamine nanoparticles. This system was designed for toxicology studies to directly expose target cells at the air-liquid-interface under *in vivo*-like conditions [31,69]. The uniformity of the particle population was confirmed by scanning electron microscopy (SEM) (Additional files 12 and 13).

Briefly, the used device (Vitrocell 6/3 CF stainless, Vitrocell Systems, Germany) contains three exposure chambers (Vitrocell module), which holds one separate insert each, allowing simultaneous exposures of 3 transwell inserts. In order to keep the cells viable, the module is equipped with a heated water jacket at a steady temperature of 37°C. For the exposure, cell culture inserts are placed into the exposure chambers containing the culture medium.

The aerosol is generated by a pneumatic nebulizer (AGF 2.0 PALAS, Karlsruhe, Germany) and is delivered through a trumpet device at a low flow rate (5 ± 0.1 mL/min/module) for a defined time of exposure to the modules.

### Statistics

Data represents the mean of at least four independent transwell inserts ± standard error of mean (SEM). Statistical analysis was done using SPSS, version 19. Statistical comparison of the means was performed by ANOVA, followed by the LSD posthoc test. In graphs, groups that are sharing the same letters are not significantly different (*P* > 0.05).

## Abbreviations

AAPH: 2,2’-azobis-2-methyl-propanimidamide-dihydrochloride; ALI: Air-liquid-interface; AM: Alveolar macrophage; CLSM: Confocal laser scanning microscopy; DAPI: 4’,6’-diamidino-2-phenylindole; DCFH-DA: Dichlorodihydrofluorescein diacetate; DCs: Dendritic cells; NPs: Nanoparticles; PM: Particulate matter; PM1: Particulate matter with aerodynamic diameter < 1 μm; PM10: Particulate matter with aerodynamic diameter < 10 μm; ROS: Reactive oxygen species; TEER: Transepithelial electrical resistance.

## Competing interests

The authors declare no conflict of interests.

## Authors' contributions

ACG, BB, LH, TS and SGK were involved in the design of the system. SGK and TS performed the CLSM experiments and the surfactant-droplet-test. TS performed the analyses of the produced cytokines. SGK performed cell viability studies as well as the experiments for permeability, TEER, and oxidative stress. ACG, BB, LH, TS and SGK contributed significantly to the interpretation of data and writing of the manuscript. All authors approved the final manuscript.

## Supplementary Material

Additional file 1 **Accessibility of resazurin depends on the pore size of the membrane.** Different cultures were exposed to cell culture medium containing 400 μM of resazurin. Both compartments, the apical and the basolateral, were filled with this solution. The conversion of resazurin on the opposite site of the cell layer was dependent on the pore size of the used transwell inserts. Data represents the mean of two independent transwell inserts ± SEM. Click here for file

Additional file 2 **Old and new version of the alveolar *in vitro* system.** A: Coculture system as proposed by Alfaro-Moreno et al. (2008). B: Variant of the system to study the potential inflammatory effects of NPs at the ALI by using a native aerosol exposure system. Adapted and modified from Klein et al. (2011). Click here for file

Additional file 3 Viability of A549 cells cultivated at the air-liquid-interface in transwell inserts with different pore sizes. A: viability after 24 h ALI; B: viability after 48 h ALI; C viability after 72 h ALI. Data represents the mean of four independent transwell inserts ± SEM. Groups that are sharing the same letters are not significantly different (*P* > 0.05). Click here for file

Additional file 4 **Transepithelial electrical resistance (TEER) of cultures grown in inserts of different pore sizes.** Electrical resistance was measured in single A549, EA.hy 926 cell cultures, cocultures of A549 and EA.hy 926 and in tetracultures to follow tightness of the cell layer in respect to the cellular composition. A: TEER measured in inserts with 0.4 μm pore size; B: TEER measured in inserts with 1 μm pore size; C: TEER measured in inserts with 3 μm pore size. Data represents the mean of two independent transwell inserts ± SEM. Click here for file

Additional file 5 **Immunohistochemistry staining of surfactant protein A in A549 cells.** A549 cells were grown in Labtek-II chambers for 48 h. Afterwards, cells were fixed, permeabilized and stained for cellular membranes, nuclei and surfactant protein A. 1: membranes stained with cell mask deep red. 2: Nuclei stained with DAPI. 3: Surfactant protein C stained with anti-surfactant-protein-A-antibody (1:200). 4: Overlay. Click here for file

Additional file 6 **Immunohistochemistry staining of surfactant protein C in A549 cells.** A549 cells were grown in Labtek-II chambers for 48 h. Afterwards, cells were fixed, permeabilized and stained for cellular membranes, nuclei and surfactant protein A. 1: membranes stained with cell mask deep red. 2: Nuclei stained with DAPI. 3: Surfactant protein C stained with anti-surfactant-protein-C-antibody (1:200). 4: Overlay. Click here for file

Additional file 7 **Surfactant droplet test.** A: A549 cell exposed for 24 h at the air-liquid-interface; B: A549 kept under submerged conditions; C: EA.hy 926 cells kept under submerged conditions. Click here for file

Additional file 8 **Immunohistochemistry staining of histamine in HMC-1 cells.** HMC-1 cells were grown over night in Labtek-II chambers coated with poly-l-lysine. Afterwards, cells were fixed, permeabilized and stained for cellular membranes, nuclei and histamine. 1: membranes stained with cell mask deep red. 2: Nuclei stained with DAPI. 3: Histamine with anti-histamine-antibody (1:200). 4: Overlay. Click here for file

Additional file 9 **Immunohistochemistry staining of CD11b receptor of differentiated and undifferentiated THP-1 cells.** THP-1 cells were differentiated over night with 20 ng/mL PMA in Labtek-II chambers **(A)** or cultivated in the absence of PMA for the same time **(B)**. Afterwards, cells were fixed and stained for cellular membranes, nuclei and CD11b. **1**: membranes stained with cell mask deep red. **2**: Nuclei stained with DAPI. **3**: CD11b receptor with anti-CD11b-antibody (1:200). **4**: Overlay. Click here for file

Additional file 10 **Seeding of endothelial and epithelial cells on inverted inserts.** EA.hy 926 endothelial cells were seeded on inverted transwell inserts (Step 1 to Step 6). First, transwell inserts were placed into a corresponding 6 well plate and the plate with the inserts was turned upside-down (Step 1). Endothelial cells were seeded on the inverted inserts and the bottom of the 6-well plate was used as lid (Step 2). Upon attachment to the basolateral side of the transwell insert, the plate with the transwell inserts was turned back to its original orientation (Step 3, 4 and 5) before the A549 cells were seeded inside the transwell (Step 6). Click here for file

Additional file 11 Vitrocell aerosol exposure system with air supply mounted on a mobile rack. Click here for file

Additional file 12 **Scanning electron microscopy image of 50 nm SiO**_**2**_**-Rhodamine nanoparticles.** Particles were sprayed onto transwell inserts to evaluate possible agglomeration or size changes using the Vitrocell™ aerosol exposure system. Inserts were exposed in the same way as transwells containing cells. Click here for file

Additional file 13 Description of methodologies used for the additional files. Click here for file
