# Supplementary figures and images for: An improved 3D tetraculture system mimicking the cellular organisation at the alveolar barrier to study the potential toxic effects of particles on the lung
Source: Part Fibre Toxicol. 2013 Jul 26;10:31. doi: 10.1186/1743-8977-10-31 (PMC3733942; doi:10.1186/1743-8977-10-31)

Inside Transwell

Outside Transwell

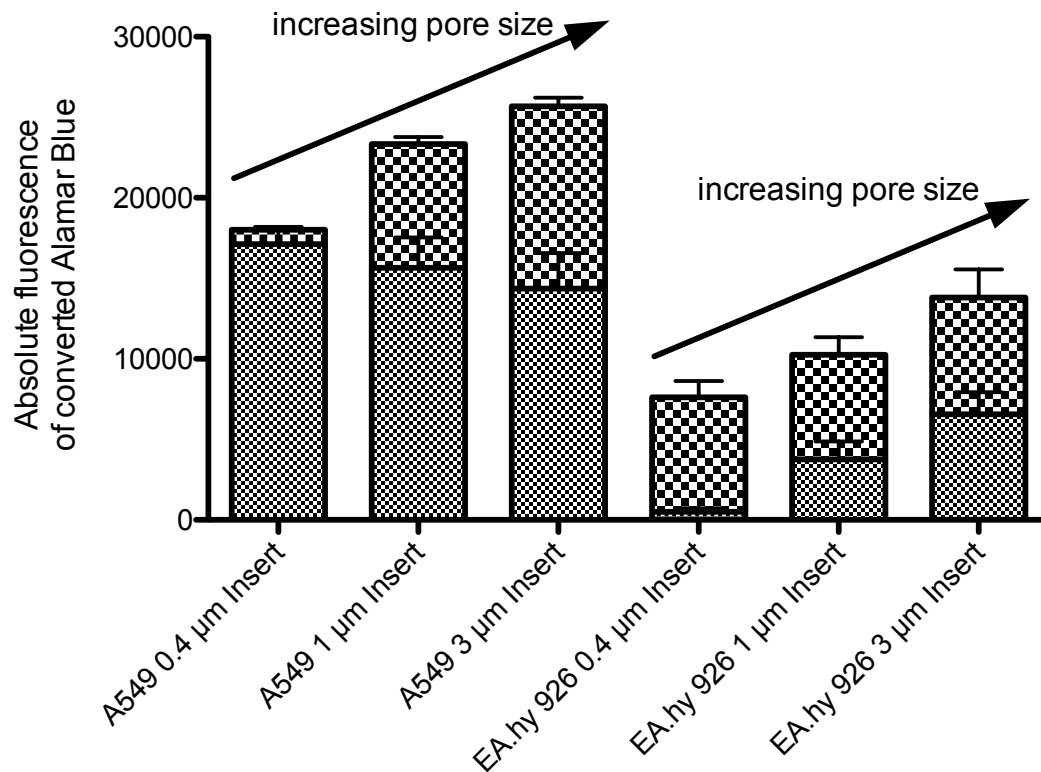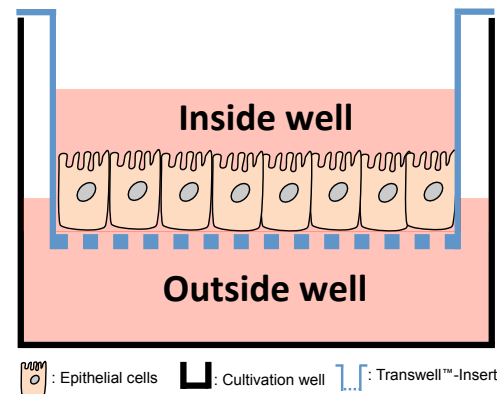

Supplement: Additional file 1 — Accessibility of resazurin depends on the pore size of the membrane. Different cultures were exposed to cell culture medium containing 400 μM of resazurin. Both compartments, the apical and the basolateral, were filled with this solution. The conversion of resazurin on the opposite site of the cell layer was dependent on the pore size of the used transwell inserts. Data represents the mean of two independent transwell inserts ± SEM. [file 1743-8977-10-31-S1.pdf]

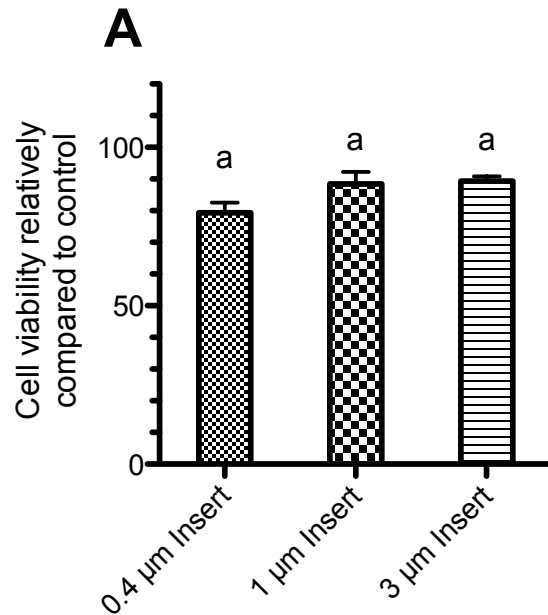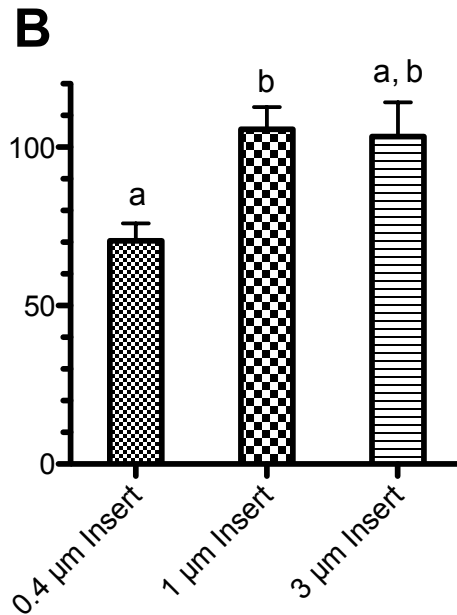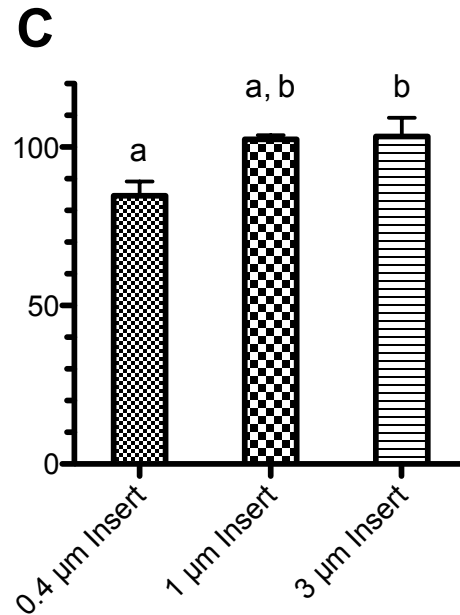

Supplement: Additional file 2 — Old and new version of the alveolar in vitro system. A: Coculture system as proposed by Alfaro-Moreno et al. (2008). B: Variant of the system to study the potential inflammatory effects of NPs at the ALI by using a native aerosol exposure system. Adapted and modified from Klein et al. (2011). [file 1743-8977-10-31-S2.pdf]

**A**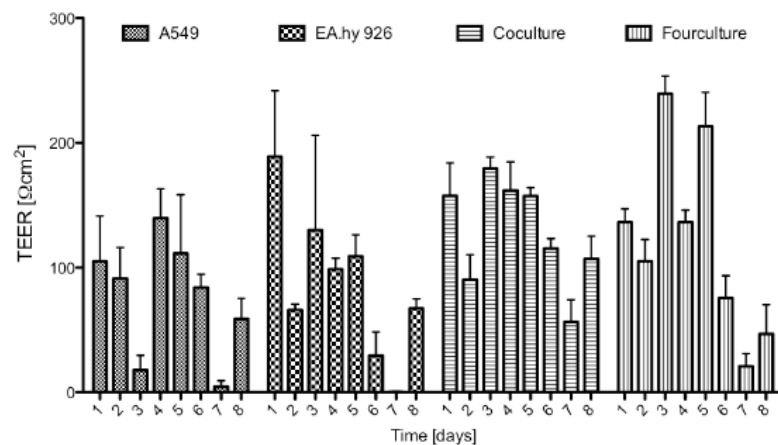**B**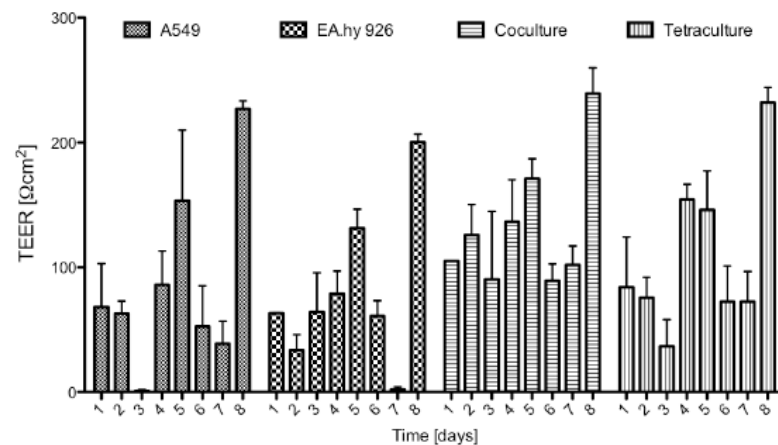**C**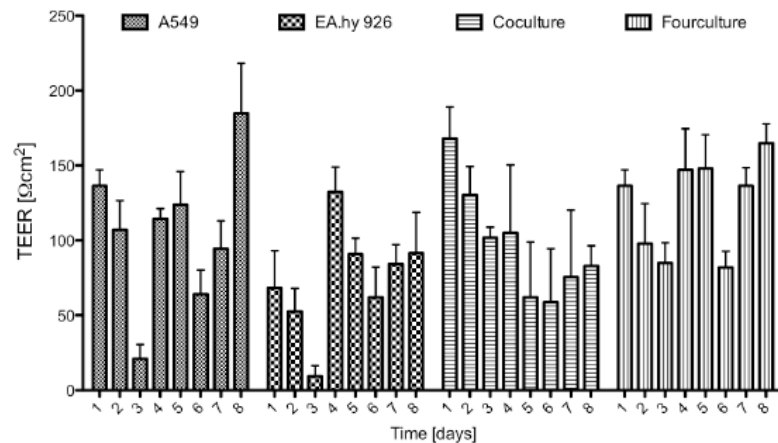

Supplement: Additional file 3 — Viability of A549 cells cultivated at the air-liquid-interface in transwell inserts with different pore sizes. A: viability after 24 h ALI; B: viability after 48 h ALI; C viability after 72 h ALI. Data represents the mean of four independent transwell inserts ± SEM. Groups that are sharing the same letters are not significantly different (P > 0.05). [file 1743-8977-10-31-S3.pdf]

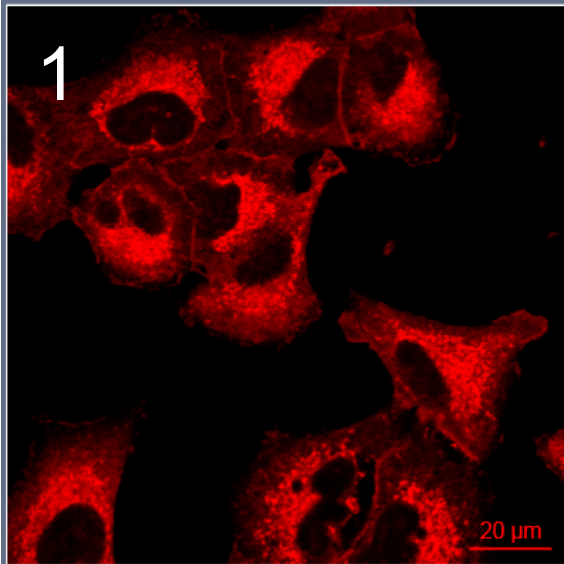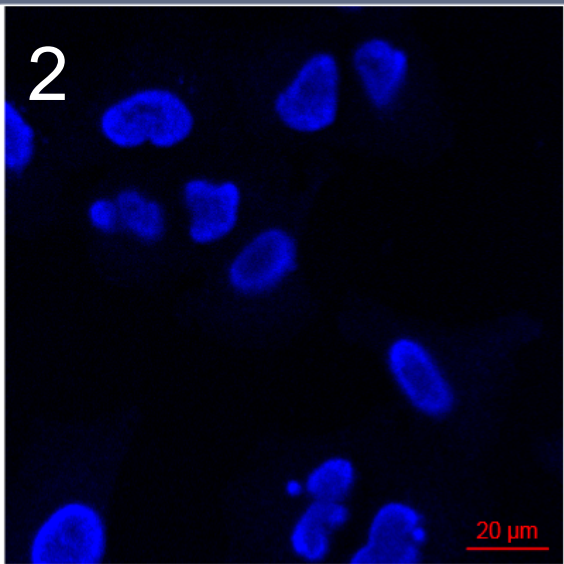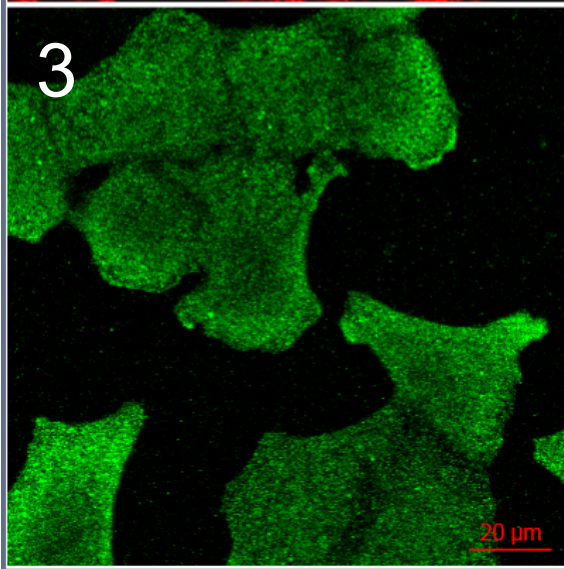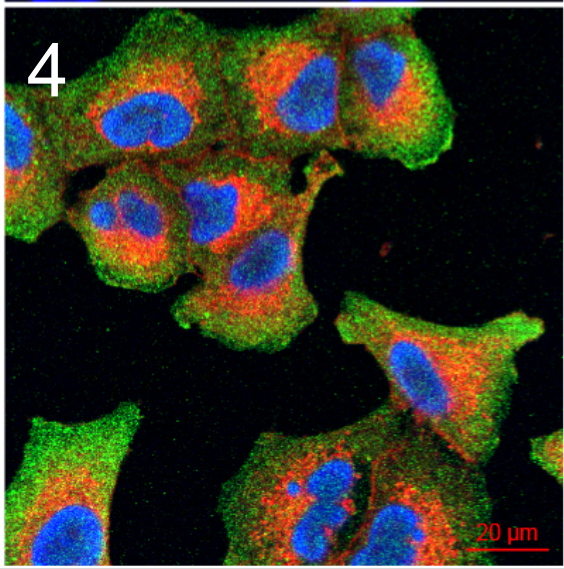

Supplement: Additional file 5 — Immunohistochemistry staining of surfactant protein A in A549 cells. A549 cells were grown in Labtek-II chambers for 48 h. Afterwards, cells were fixed, permeabilized and stained for cellular membranes, nuclei and surfactant protein A. 1: membranes stained with cell mask deep red. 2: Nuclei stained with DAPI. 3: Surfactant protein C stained with anti-surfactant-protein-A-antibody (1:200). 4: Overlay. [file 1743-8977-10-31-S5.pdf]

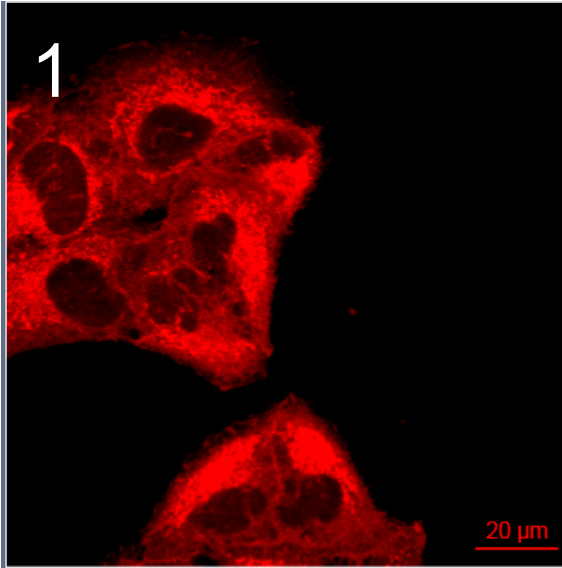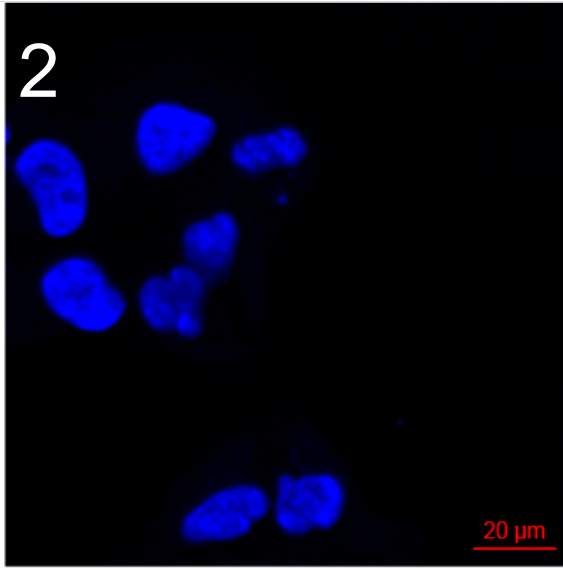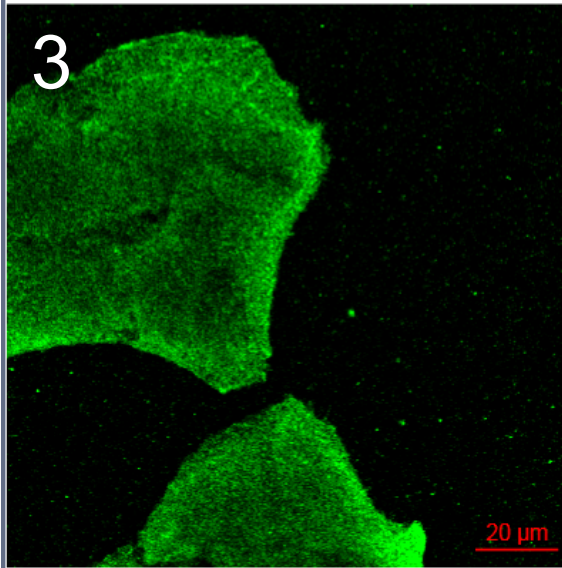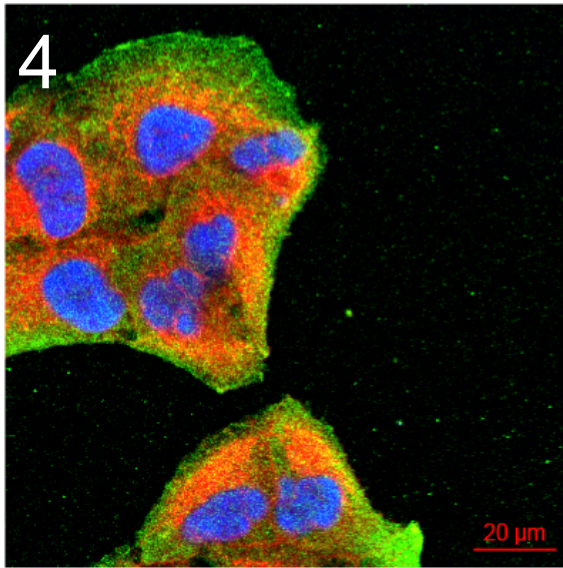

Supplement: Additional file 6 — Immunohistochemistry staining of surfactant protein C in A549 cells. A549 cells were grown in Labtek-II chambers for 48 h. Afterwards, cells were fixed, permeabilized and stained for cellular membranes, nuclei and surfactant protein A. 1: membranes stained with cell mask deep red. 2: Nuclei stained with DAPI. 3: Surfactant protein C stained with anti-surfactant-protein-C-antibody (1:200). 4: Overlay. [file 1743-8977-10-31-S6.pdf]

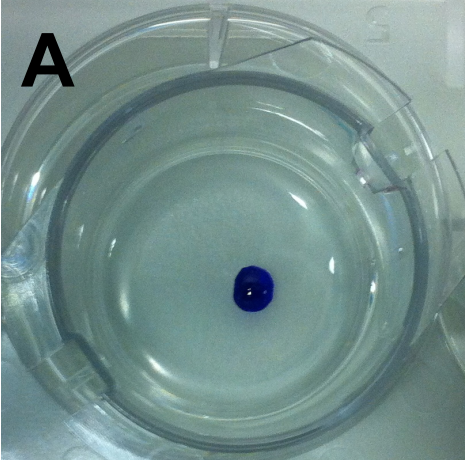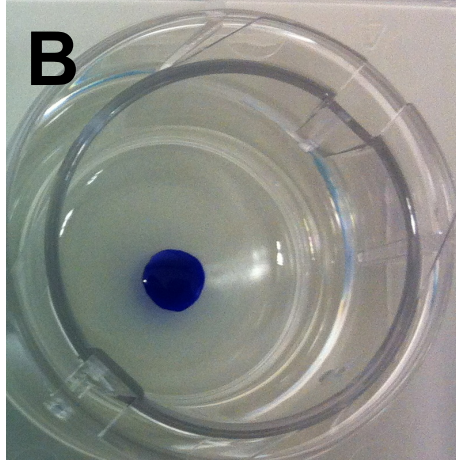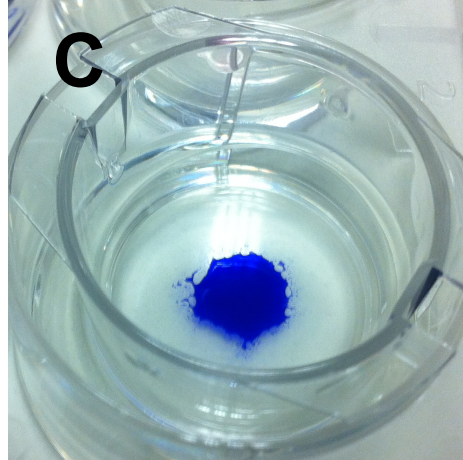

Supplement: Additional file 7 — Surfactant droplet test. A: A549 cell exposed for 24 h at the air-liquid-interface; B: A549 kept under submerged conditions; C: EA.hy 926 cells kept under submerged conditions. [file 1743-8977-10-31-S7.pdf]

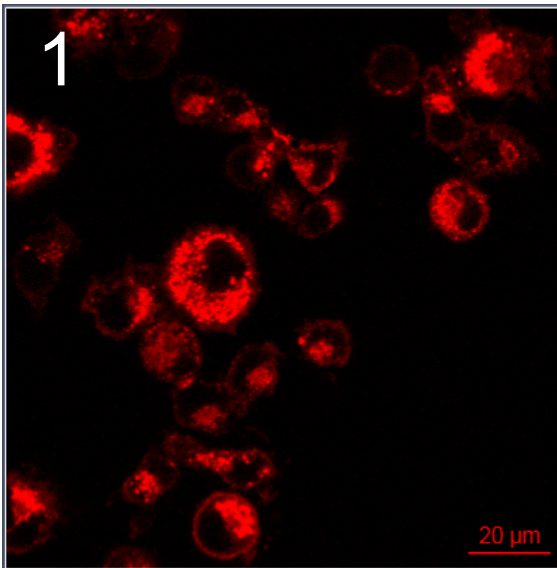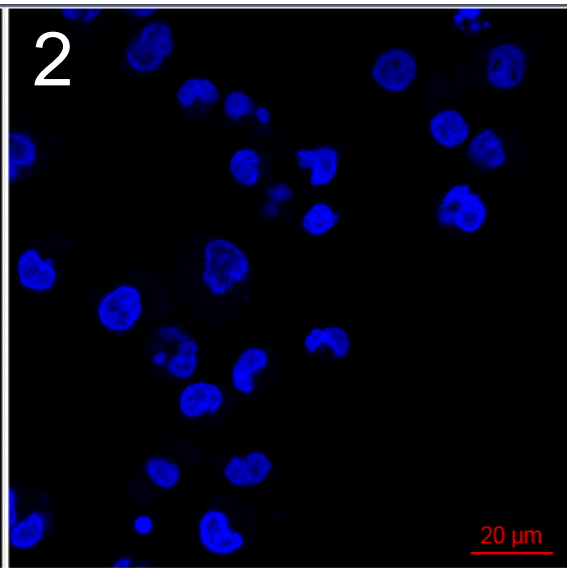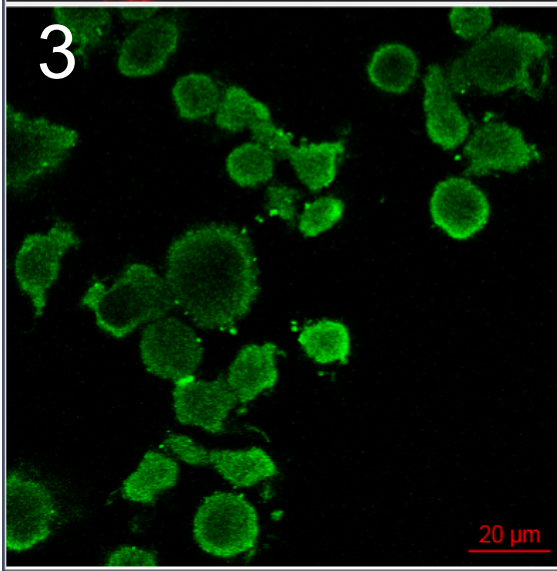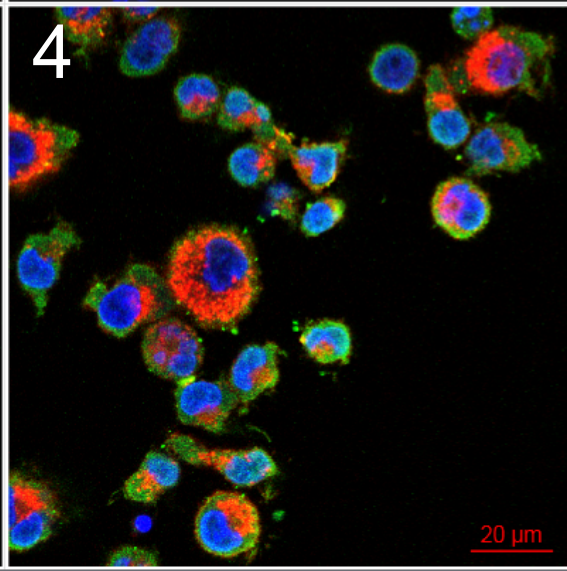

Supplement: Additional file 8 — Immunohistochemistry staining of histamine in HMC-1 cells. HMC-1 cells were grown over night in Labtek-II chambers coated with poly-l-lysine. Afterwards, cells were fixed, permeabilized and stained for cellular membranes, nuclei and histamine. 1: membranes stained with cell mask deep red. 2: Nuclei stained with DAPI. 3: Histamine with anti-histamine-antibody (1:200). 4: Overlay. [file 1743-8977-10-31-S8.pdf]

**A**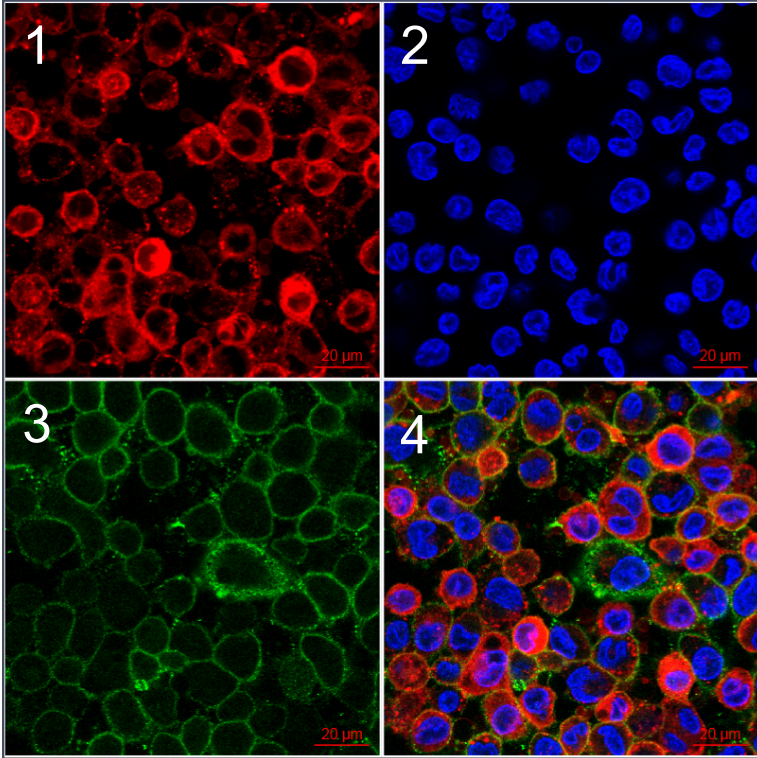**B**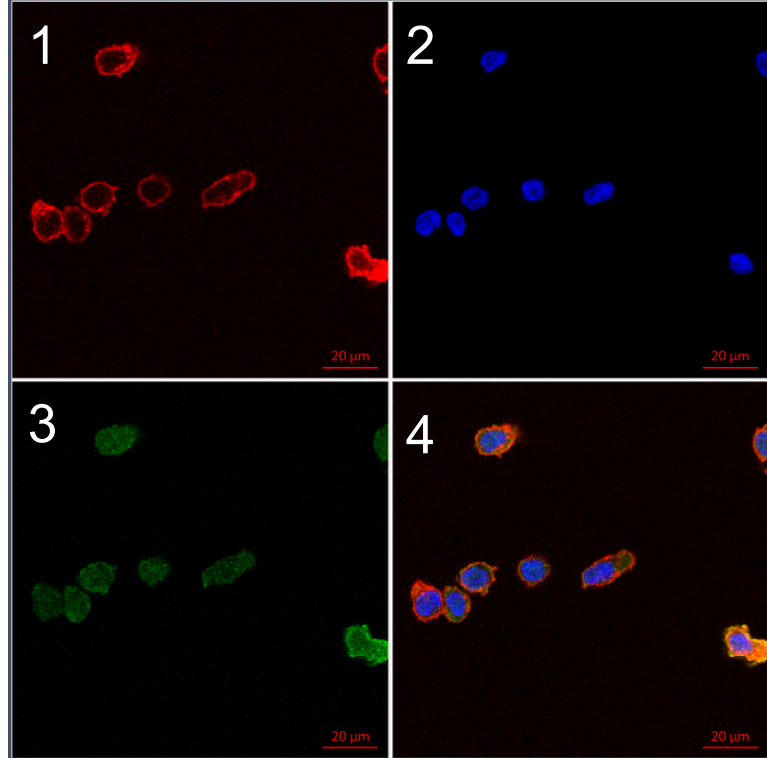

Supplement: Additional file 9 — Immunohistochemistry staining of CD11b receptor of differentiated and undifferentiated THP-1 cells. THP-1 cells were differentiated over night with 20 ng/mL PMA in Labtek-II chambers (A) or cultivated in the absence of PMA for the same time (B). Afterwards, cells were fixed and stained for cellular membranes, nuclei and CD11b. 1: membranes stained with cell mask deep red. 2: Nuclei stained with DAPI. 3: CD11b receptor with anti-CD11b-antibody (1:200). 4: Overlay. [file 1743-8977-10-31-S9.pdf]

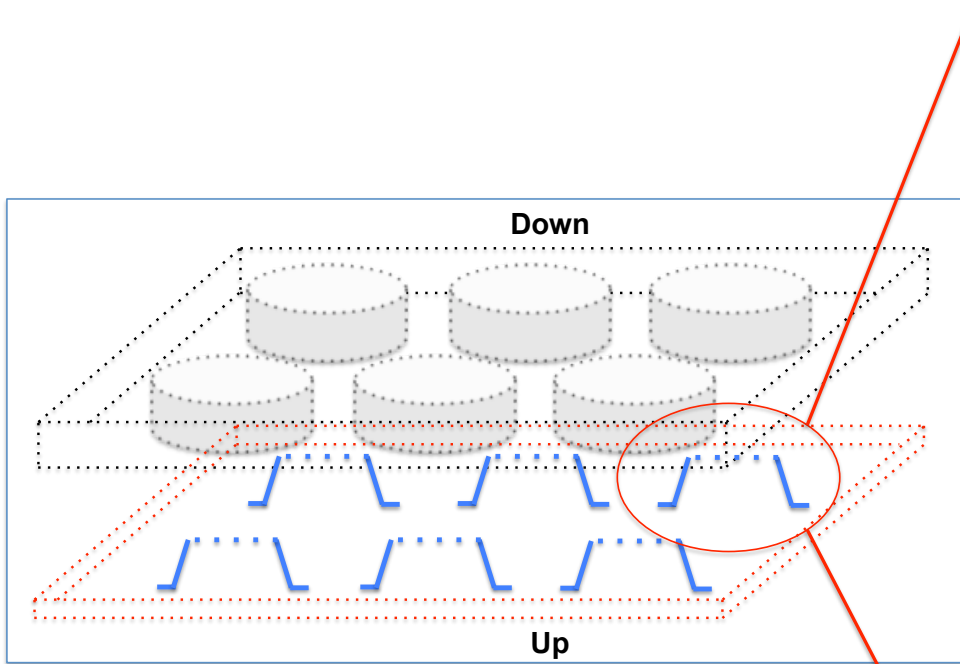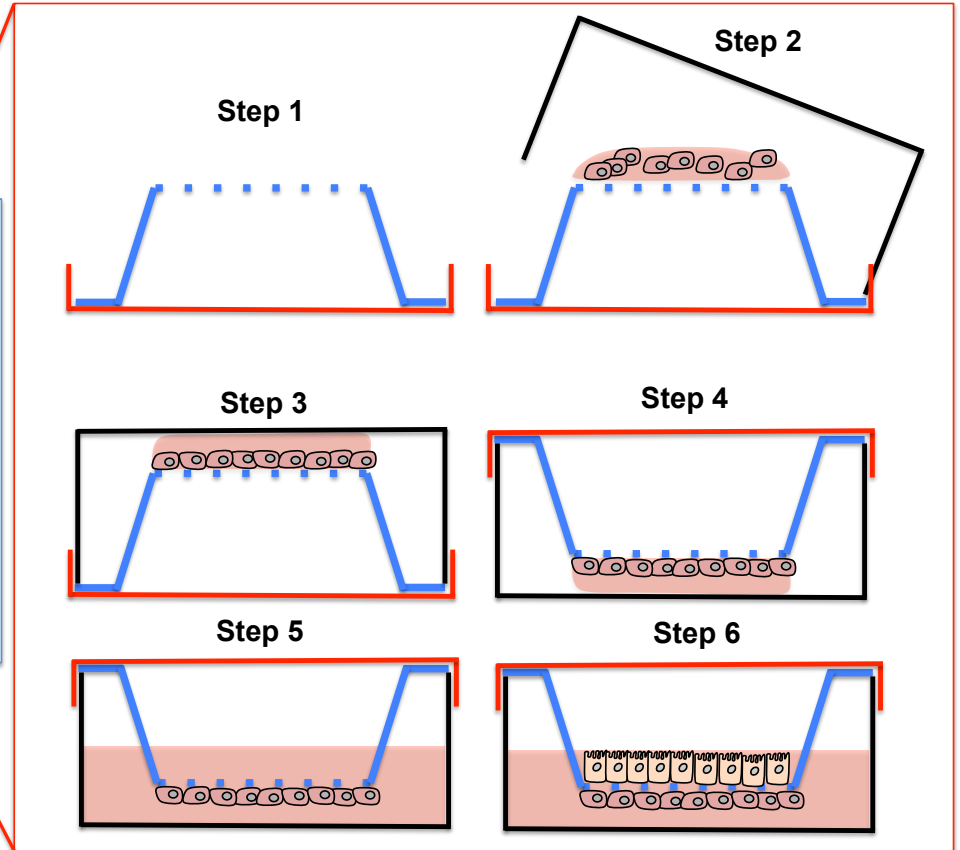

Supplement: Additional file 10 — Seeding of endothelial and epithelial cells on inverted inserts. EA.hy 926 endothelial cells were seeded on inverted transwell inserts (Step 1 to Step 6). First, transwell inserts were placed into a corresponding 6 well plate and the plate with the inserts was turned upside-down (Step 1). Endothelial cells were seeded on the inverted inserts and the bottom of the 6-well plate was used as lid (Step 2). Upon attachment to the basolateral side of the transwell insert, the plate with the transwell inserts was turned back to its original orientation (Step 3, 4 and 5) before the A549 cells were seeded inside the transwell (Step 6). [file 1743-8977-10-31-S10.pdf]

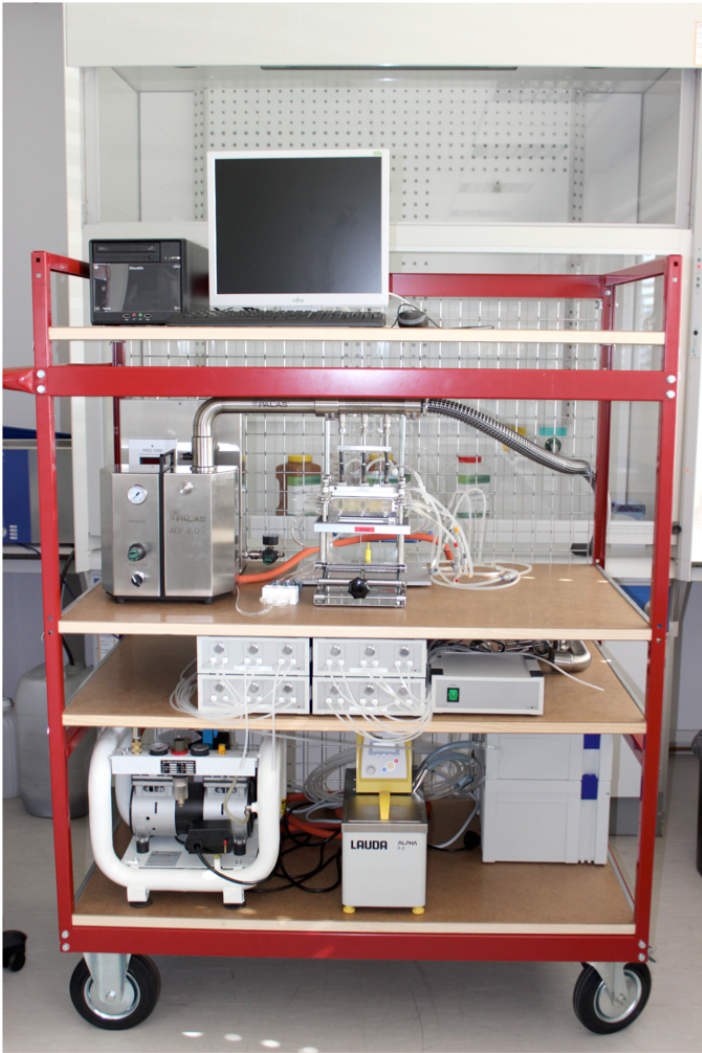

Supplement: Additional file 11 — Vitrocell aerosol exposure system with air supply mounted on a mobile rack. [file 1743-8977-10-31-S11.pdf]

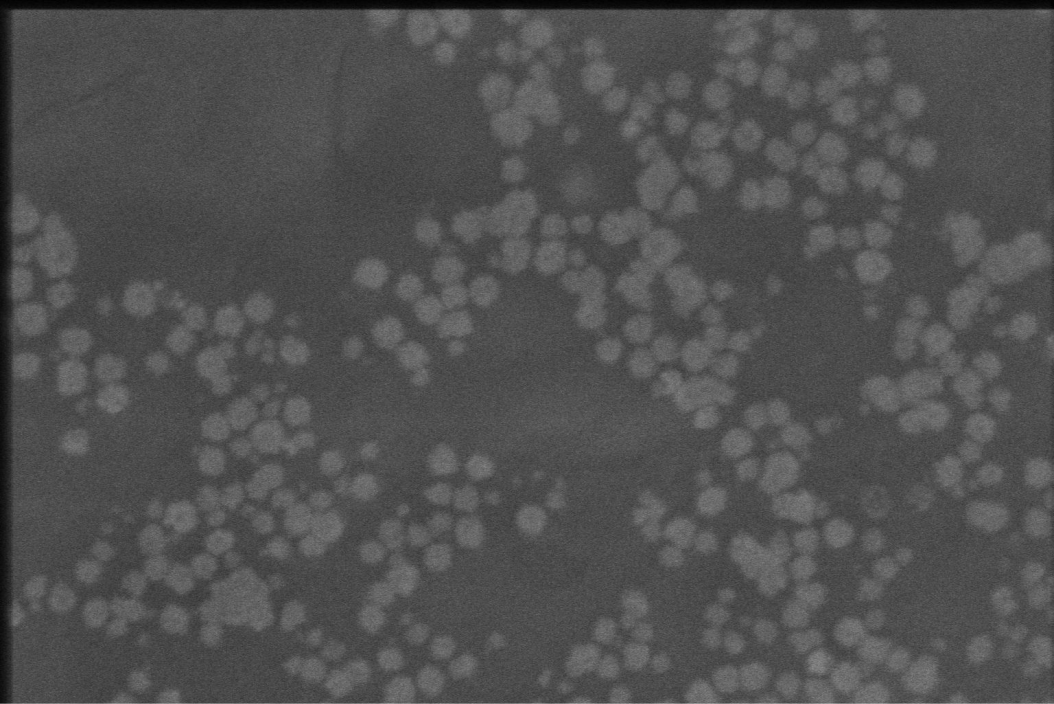

|                                                                                  |                         |               |            |              |                                                                                                   |            |               |             |                                                                                            |  |
|----------------------------------------------------------------------------------|-------------------------|---------------|------------|--------------|---------------------------------------------------------------------------------------------------|------------|---------------|-------------|--------------------------------------------------------------------------------------------|--|
| 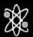 | 5/15/2013<br>5:39:41 PM | HV<br>3.00 kV | det<br>TLD | WD<br>5.1 mm | mag 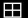<br>65 000 x | mode<br>SE | curr<br>25 pA | tilt<br>0 ° | 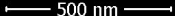 500 nm |  |
|                                                                                  |                         |               |            |              |                                                                                                   |            |               |             | Helios LIPPMAN                                                                             |  |

Supplement: Additional file 12 — Scanning electron microscopy image of 50 nm SiO2-Rhodamine nanoparticles. Particles were sprayed onto transwell inserts to evaluate possible agglomeration or size changes using the Vitrocell™ aerosol exposure system. Inserts were exposed in the same way as transwells containing cells. [file 1743-8977-10-31-S12.pdf]
