# Supplementary material for: An improved 3D tetraculture system mimicking the cellular organisation at the alveolar barrier to study the potential toxic effects of particles on the lung
Source: Part Fibre Toxicol. 2013 Jul 26;10:31. doi: 10.1186/1743-8977-10-31 (PMC3733942; doi:10.1186/1743-8977-10-31)
Supplement: Additional file 4 — Transepithelial electrical resistance (TEER) of cultures grown in inserts of different pore sizes. Electrical resistance was measured in single A549, EA.hy 926 cell cultures, cocultures of A549 and EA.hy 926 and in tetracultures to follow tightness of the cell layer in respect to the cellular composition. A: TEER measured in inserts with 0.4 μm pore size; B: TEER measured in inserts with 1 μm pore size; C: TEER measured in inserts with 3 μm pore size. Data represents the mean of two independent transwell inserts ± SEM. [file 1743-8977-10-31-S4.pdf]

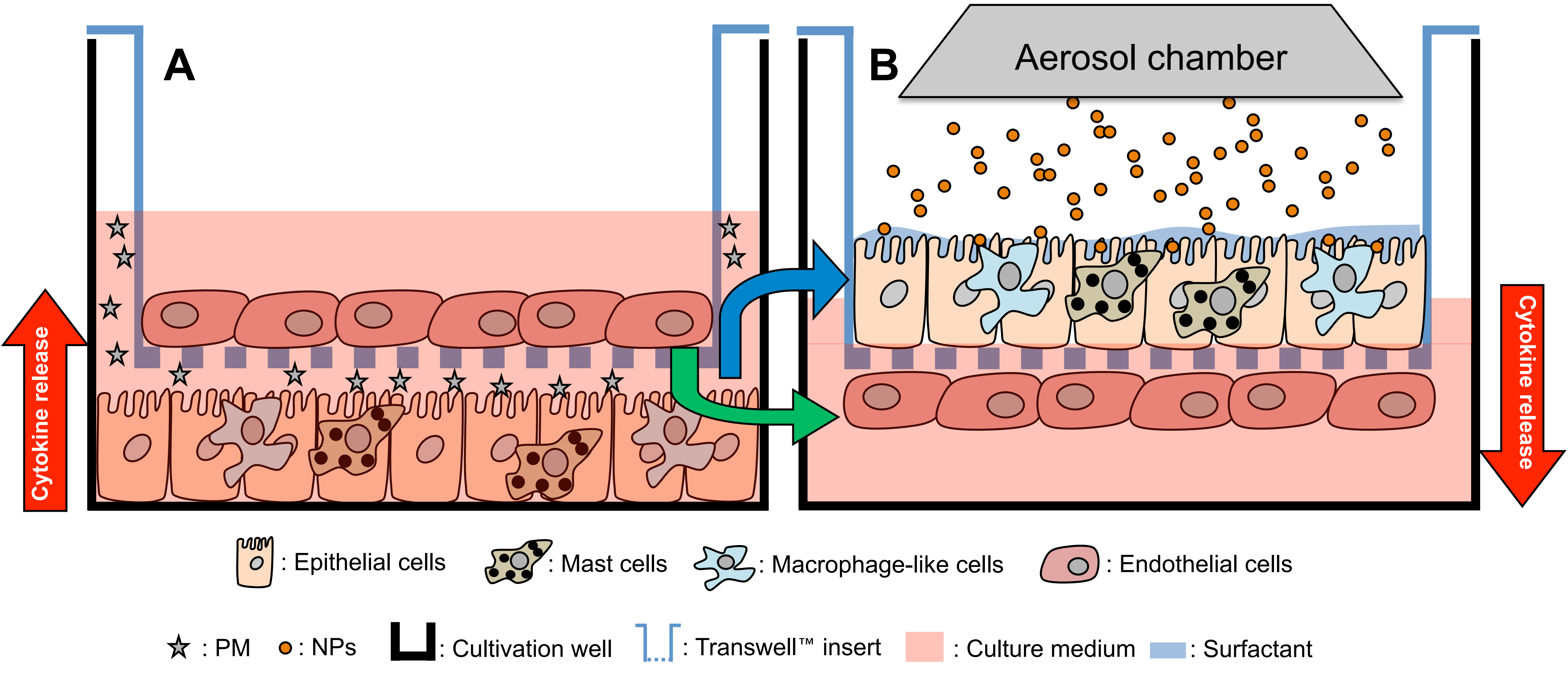

A
B
Aerosol chamber
Cytokine release
Cytokine release
: Epithelial cells
: Mast cells
: Macrophage-like cells
: Endothelial cells
: PM
: NPs
: Cultivation well
: Transwell™ insert
: Culture medium
: Surfactant
