# Supplementary material for: An improved 3D tetraculture system mimicking the cellular organisation at the alveolar barrier to study the potential toxic effects of particles on the lung
Source: Part Fibre Toxicol. 2013 Jul 26;10:31. doi: 10.1186/1743-8977-10-31 (PMC3733942; doi:10.1186/1743-8977-10-31)
Supplement: Additional file 13 — Description of methodologies used for the additional files. [file 1743-8977-10-31-S13.pdf]

Supplementary material

MATERIAL AND METHODS

Surfactant droplet test

Surfactant droplet test was performed as described by [1]. Briefly 549 cells were cultured in submersion on insert membranes. The medium was then removed from the apical compartment well and the cells were cultivated at the air-liquid-interface for 24 h. In order to determine the surface tension, DMP/O droplets were placed on the cell surface. Droplet diameters, d0 and d, were measured before and after deposition. A large diameter indicates a high surface tension, e.g. of cells grown under submerged conditions. A small diameter of the drop indicates a lower surface tension, e.g. of cells grown under ALI conditions with the possibility to secrete surfactant.

Transepithelial Electrical Resistance Measurements

Transepithelial electrical resistance (TEER) was measured with the Millicell-ERS system (MERS 000 01; Millipore AG, Volketswil, Switzerland). The mean of three measurements per insert was determined. The electrical resistance of inserts without cells was subtracted from all samples, and the resistance values were multiplied with the surface area of the inserts (4.2 cm^2). Electrical resistance was measured in single A549, EA.hy 926 cell cultures, cocultures of A549 and EA.hy 926 and in tetracultures to follow tightness of the cell layer in respect to the cellular composition.

Adaption of the resazurin metabolism assay for the 3D tetraculture

Transwell inserts were washed twice with PBS. Afterwards, 2 mL of medium containing 400 µM resazurin were added to the upper and the lower compartment. Cells were incubated for

one hour at 37 °C and 5% CO_2. After the incubation, aliquots of 500 µL were taken from the upper and the lower compartment and transferred into a 12 well plate to measure separately the fluorescence for both compartments (inside and outside of the transwell insert). Fluorescence reading was done with a multi-mode microplate reader (ex: 530 nm, em: 590 nm; Biotek, Germany).

Scanning electron microscopy (SEM)

The Vitrocell aerosol exposure device (Vitrocell, Taufkirchen, Germany) (Additional file 10) was used for dynamic delivery and exposure of cells to aerosolized PBS and to PBS containing 50 nm SiO_2-Rhodamine nanoparticles. Transwells inserts were exposed for 30 min as described for transwell inserts containg the tetraculture system. Afterwards samples were metallised with a 20 nm golf film under vacuum. Scanning electron microscopy was done with a FIB-SEM (FEI, Eindhoven, The Netherlands) at 25 kV and 25 mA.

References

1. Rothen-Rutishauser B, Blank F, Mühlfeld C, Gehr P: ***In vitro* models of the human epithelial airway barrier to study the toxic potential of particulate matter.** *Expert Opin Drug Metab Toxicol* 2008, **4:**1075-1089.
